# spCLUE: a contrastive learning approach to unified spatial transcriptomics analysis across single-slice and multi-slice data

**DOI:** 10.1186/s13059-025-03636-0

**Published:** 2025-06-23

**Authors:** Xiang Wang, Wei Vivian Li, Hongwei Li

**Affiliations:** 1 School of Mathematics and Physics, China University of Geosciences, Wuhan, China; 2 Department of Statistics, University of California, Riverside, USA

## Abstract

Advances in spatial transcriptomics demand new tools to integrate data across tissue slices and identify consistent spatial domains. We introduce spCLUE, a comprehensive framework combining multi-view graph network, contrastive learning, attention mechanisms, and a batch prompting module to learn informative spot representations and integrate data from both aligned and unaligned samples. spCLUE outperforms nine single-slice and seven multi-slice methods when tested on diverse datasets and reveals biologically relevant domains across different tissues and conditions. spCLUE offers a powerful solution to spatial domain analysis and integration in spatial transcriptomics, enabling more accurate and interpretable studies of tissue organization.

**Supplementary information:**

The online version contains supplementary material available at 10.1186/s13059-025-03636-0.

## Background

With advancements in sequencing technologies, spatially resolved transcriptomics (SRT) has gained significant popularity for its ability to measure gene expression profiles while preserving the spatial organization of cells [1–3]. A variety of SRT methods, such as 10x Visium [4], Slide-seqV2 [5], Stereo-seq [6], and BaristaSeq [7], can capture gene expression profiles at spatial locations (i.e., spots) while retaining their spatial coordinates. A key application of SRT data is the identification of spatial domains, also known as spatial clusters, which are groups of cells or spots exhibiting similar gene expression patterns [8, 9]. Identifying these domains is critical for uncovering biological insights, as spatial domains often correspond to functional tissue units, such as cell types, microenvironments, or pathological regions. By integrating spatial and transcriptomic information, SRT provides a more comprehensive view of the spatial organization of tissues, facilitating discoveries that may not be possible with gene expression data alone [10].

Clustering methods for SRT data can be broadly categorized into two groups based on how spatial locations are utilized: non-spatial methods and spatial methods. Non-spatial methods are standard approaches that rely solely on gene expression data for clustering. For example, Seurat [11] applies the Louvain algorithm to low-dimensional data obtained via principal component analysis [12] to generate clustering results. Spatial methods, on the other hand, integrate spatial locations with gene expression data to infer spatial clusters. For instance, Giotto [13] uses a Markov random field model to combine spatial priors with gene expression profiles for such inference. BayesSpace [14] incorporates spatial priors to encourage physically proximate cells to belong to the same cluster. BASS [15] simultaneously performs cell-type clustering and spatial domain segmentation using a Bayesian hierarchical model. In addition, a growing number of deep-learning-based methods have been developed to decipher spatial domains, many of which leverage graph neural networks (GNNs) [16]. For example, SpaGCN [17] combines spatial locations and histology data to construct an undirected weighted graph, integrating a graph convolutional network (GCN) [18] with a deep embedded clustering framework [19] to identify spatial domains. STAGATE [20] constructs a spatial neighbor graph and trains a graph attention auto-encoder network [21] to delineate spatial domains. CCST [22] and SpaceFlow [23] utilize the deep graph infomax framework [24] with tailored designs to cluster SRT data. SEDR [25] employs a variational graph auto-encoder [26] with a masked self-supervised learning mechanism to learn spot representations, followed by clustering to infer spatial domains. GraphST [27] utilizes graph contrastive learning to embed gene expression profiles and spatial similarity into a latent representation space, which is used for spatial clustering. stDyer [28] combines a Gaussian mixture variational auto-encoder with a graph attention network to achieve representation learning and clustering of SRT data.

With the growing adoption of SRT methods, spatial transcriptomics data increasingly consist of multiple slices of samples derived from the same or different subjects. Analyzing each slice independently can hinder unbiased comparisons between samples or subjects, making it essential to identify spatial domains simultaneously across multiple slices. Non-spatial methods, such as Harmony [29], have been utilized for correcting batch effects in single-cell expression data and hold potential for multi-slice clustering in SRT datasets [15]. Additionally, several spatial methods have been adapted to handle multi-slice data. For example, STAGATE and GraphST extend their networks to accommodate multi-slice datasets under the assumption that spatial coordinates across slices are already aligned. In contrast, methods like BayesSpace, BASS, and SEDR can perform spatial domain identification for both aligned and unaligned slices, offering greater flexibility in handling complex datasets. Furthermore, STAligner, an extension of the STAGATE method [30], integrates multiple slices by defining mutual nearest neighbors in the latent space, enabling analysis of spatial domains across slices without prior alignment.

In this work, we introduce spCLUE (spatial Contrastive Learning for Unified Embeddings), a unified framework designed to analyze both single-slice and multi-slice spatial transcriptomics data. spCLUE leverages a graph-contrastive-learning paradigm to infer spatial domains and spot representations, addressing several key challenges and limitations in spatial transcriptomics analysis. First, most existing spatial domain identification methods either ignore spatial information or represent it through a single graph that combines spatial coordinates and gene expression. This often involves predefined weighting schemes or using gene expression as node features on spatial graphs, which may oversimplify the relationship between spatial proximity and transcriptional similarity, and limit the model’s ability to capture complementary signals from each view. To overcome these limitations, spCLUE employs a multi-view graph learning strategy that constructs separate graphs for spatial and gene expression data. This allows the model to extract distinct yet complementary insights from each view. Unlike prior multi-view methods such as Spatial-MGCN [31] and MuCoST [32], which are limited to single-slice analyses, spCLUE is designed for both single-slice and multi-slice analysis. It integrates the learned representations using a novel attention module, providing a flexible and adaptive framework that improves spatial domain identification without relying on ad hoc graph fusion strategies. Second, existing deep learning methods for spatial transcriptomics often focus on learning spot embeddings that capture biological variation, but they do not directly support clustering for spatial domain identification. While contrastive learning has been used to improve embedding quality [22, 32, 33], prior approaches typically compare real versus corrupted graphs or rely on predefined negative samples, which may enhance instance-level representations but do not explicitly encourage a clustering structure. To address this, spCLUE introduces a novel contrastive learning framework that promotes both biological coherence and cluster separation. It combines instance-level contrastive learning, which aligns spot representations across spatial and gene expression views, with a clustering-level module that encourages the formation of distinct clusters without requiring explicit clustering during training. This dual strategy aligns representation learning with the downstream goal of spatial domain discovery. Third, for multi-slice spatial transcriptomics analysis, many existing methods do not explicitly model batch effects. Some rely on aligned slices without incorporating batch correction into the model, while others depend on external preprocessing tools originally developed for scRNA-seq data. However, treating batch correction as a preprocessing step risks the loss of biologically meaningful variation, which cannot be recovered during downstream analysis. To address this, spCLUE introduces a batch prompting module that explicitly learns batch-specific embeddings during training and removes them prior to learning spot embeddings. This design enables the model to isolate and discard technical variation early in the pipeline, allowing the final embeddings to more accurately reflect biologically relevant spatial structure. The key features of spCLUE in comparison to alternative methods are summarized in Additional file 1: Table S1. We evaluated the performance of spCLUE on six benchmark datasets generated by different platforms, including 10x Visium, Slide-seqV2, Stereo-seq, and BaristaSeq. Comparative analyses demonstrated that spCLUE achieves consistently high performance in spatial clustering tasks for both single-slice and multi-slice data.

## Results

### Overview of the spCLUE method

The spCLUE method employs a graph convolutional network framework integrated with graph contrastive learning to derive latent embeddings and identify spatial domains (clusters) from single-slice or multi-slice spatial transcriptomics data. We first define this computational problem in the general context of multiple slices. Suppose there are *L* slices of spatial transcriptomics data, where each slice has been preprocessed to produce a gene expression matrix and a spatial location matrix. These matrices are denoted as $\mathcal {D} = \{\varvec{\textrm{X}}_1,\dots , \varvec{\textrm{X}}_L\}\cup \{\varvec{\textrm{S}}_1,\dots ,\varvec{\textrm{S}}_L\}$, where $\varvec{\textrm{X}}_l\in \mathbb {R}^{m_l\times n}$ is the *l-*th expression matrix with $m_l$ spots and *n* genes and $\varvec{\textrm{S}}_l\in \mathbb {R}^{m_l\times 2}$ is the corresponding spatial location matrix. For the *l-*th slice, we use $\varvec{\textrm{x}}_i^{(l)}$ to denote the gene expression vector of the *i-*th spot and $\varvec{s}_i^{(l)}$ represents the corresponding spatial location of this spot. The objective of spCLUE is to find a joint embedding mapping $f:\mathbb {R}^n\times \mathbb {R}^2 \rightarrow \mathbb {R}^d\ (d\ll n)$ to project all spots into a lower-dimensional embedding space. Then, the spot embeddings are extracted as

1 $$\begin{aligned} \mathcal {Z}=\left\{ \varvec{z}_i^{(l)}|\varvec{z}_i^{(l)} = f\left( \varvec{\textrm{x}}_i^{(l)}, \varvec{s}_i^{(l)}\right) ;i=1,2,\cdots ,m_l, l=1,2,\cdots , L\right\} , \end{aligned}$$

where $\varvec{z}_i^{(l)}$ represents the embedding of the *i*th spot in slice *l*. For the identification of spatial clusters, we need to obtain a partition $\mathscr {T} =\left\{ \left. \mathcal {C}_j\right| j=1,2,\cdots ,K\right\}$, such that $\mathcal {Z} = \mathcal {C}_1\cup \mathcal {C}_2\cup \cdots \cup \mathcal {C}_K$ and $\mathcal {C}_i \cap \mathcal {C}_j = \varnothing \ (i\not = j)$, where $\mathcal {C}_j$ represents cluster *j*.

**Fig. 1:**
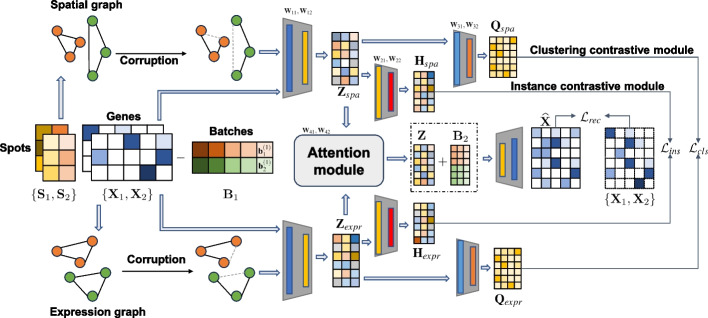
Overview of the spCLUE model. Taking a two-slice dataset as an example, spCLUE begins by constructing a multi-view graph (spatial view and expression view) for each slice. Next, it extracts spot representations through a graph contrastive learning framework, incorporating a batch prompting module, a clustering contrastive module, and an instance contrastive module. Finally, an attention module integrates the spot embeddings learned from the two contrastive modules, and the decoder reconstructs the gene expression profiles

To effectively integrate information from both gene expression data and spatial locations, spCLUE employs a multi-view graph construction approach (Fig. 1; see “Methods” for details). For a single tissue slice, spCLUE first constructs two separate graphs: one based on spatial locations and the other on gene expression data. From the spatial location matrix $\textbf{S} \in \mathbb {R}^{m\times 2}$, spCLUE computes an adjacency matrix $\varvec{\textrm{A}}_{spa}\in \mathbb {R}^{m\times m}$ using Euclidean distances. Similarly, for the gene expression matrix $\varvec{\textrm{X}}\in \mathbb {R}^{m\times n}$, spCLUE generates an adjacency matrix $\varvec{\textrm{A}}_{expr}\in \mathbb {R}^{m\times m}$ based on Pearson correlations between spots. Using the multi-view graph, spCLUE employs an encoder-decoder framework to train the network. Each graph $\varvec{\textrm{A}}_{*} \in \{\varvec{\textrm{A}}_{spa}, \varvec{\textrm{A}}_{expr}\}$ is processed through a GCN-based encoder to derive spot embeddings $\varvec{\textrm{Z}}_{*}$ in the latent space. A graph contrastive learning module, which considers both instance-level and cluster-level similarities, aligns the embeddings from the two views, promoting consistency between them. Subsequently, an attention layer integrates the two embeddings to generate unified spot representations $\varvec{\textrm{Z}}$. These fused representations are then fed into a decoder to reconstruct the gene expression matrix $\widehat{\textbf{X}}$. After training, the fused representations produced by the attention layer are used for clustering analysis to identify spatial domains. For multi-slice spatial transcriptomics data, we extend the multi-view graph construction approach in spCLUE to handle multiple slices before applying the graph contrastive learning module. Additionally, spCLUE incorporates a batch prompting module to learn batch embeddings, which are subsequently removed to enhance the integration of multi-slice data.

Below, we present an evaluation of spCLUE’s performance using six real datasets obtained from various spatially resolved transcriptomic technologies and tissue locations (Additional file 1: Table S2 and Additional file 1: Supplementary Methods). To benchmark spCLUE in the task of single-slice analysis, we compared its performance against nine alternative methods for spatial domain detection: Seurat [11], BayesSpace [14], BASS [15], SpaceFlow [23], SpaGCN [17], CCST [22], STAGATE [20], GraphST [27], and SEDR [25]. Additional file 1: Table S1 summarizes the key features of these methods, and their implementation details are presented in Additional file 1: Supplementary Methods. Among these, Seurat is a widely used single-cell clustering method that relies solely on gene expression data without using any spatial information, whereas the other eight methods incorporate spatial information. Additionally, five of these nine methods are designed to be able to directly handle multi-slice data, including BayesSpace, BASS, STAGATE, GraphST, and SEDR. Notably, STAGATE and GraphST are suitable only for datasets with aligned spatial coordinates, so their results on unaligned slices were not presented. For the task of multi-slice integration, we also compared spCLUE with the Harmony [29] and STAligner [30] methods.

### spCLUE improves spatial domain identification on the DLPFC dataset

**Fig. 2:**
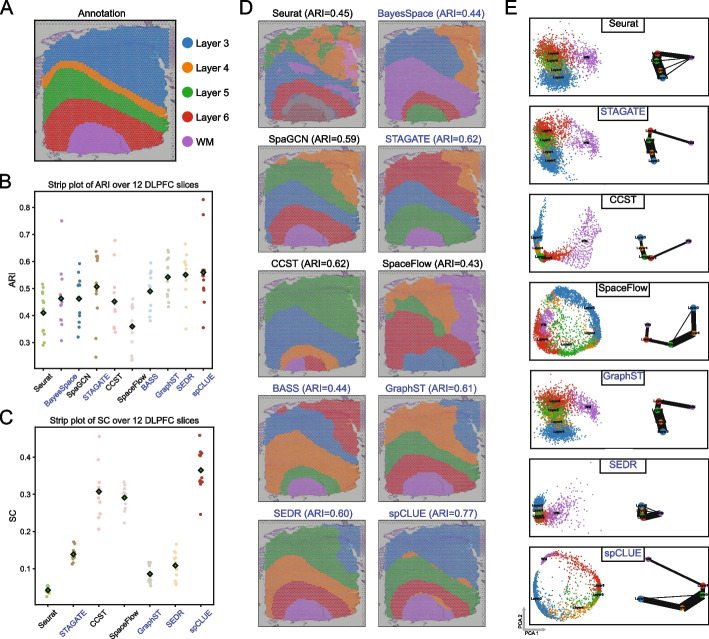
Spatial domain identification on single slices of the DLPFC dataset. **A** Annotation of cortical layers and white matter for spots in an example slice (slice 151672). **B** ARI scores of the ten methods across the 12 DLPFC slices. The circles denote ARI scores on individual slices, and the diamonds mark the average ARI score across the 12 slices. The methods are ordered based on publication year, and methods applicable to multi-slice data are highlighted with the blue color. **C** SC values of the ten methods across the 12 DLPFC slices. **D** Spatial domain labels obtained by the ten methods on slice 151672. **E** PCA visualizations and PAGA graphs obtained by representation learning methods on slice 151672. Edge thickness in PAGA graph indicates connectivity between spot groups

We first applied spCLUE and alternative methods to a human dorsolateral prefrontal cortex (DLPFC) dataset [4], which is widely used for benchmarking spatial clustering approaches. This dataset comprises 12 slices, with spots annotated as belonging to seven distinct layers, including the white matter (WM) and cortical layers 1 through 6 (Fig. 2A). To evaluate performance on single-slice data, we applied all methods independently to each slice. The identified spatial domains were then assessed using the adjusted Rand index (ARI) [34] and normalized mutual information (NMI) [35], both of which are external evaluation metrics that assess how well the predicted clusters align with known spatial domain annotations (Additional file 1: Supplementary Methods). Based on both ARI (Fig. 2B) and NMI (Additional file 1: Fig. S1A) scores, spCLUE was among the top three methods. In particular, it achieved the highest average ARI among all methods, followed by SEDR and GraphST (Fig. 2B). Focusing on methods capable of generating low-dimensional spot embeddings, we further evaluated these methods using the silhouette coefficient (SC) [36] and the Calinski-Harabasz (CH) index [37], which are internal metrics focused on evaluating the overall clustering structure itself, rather than how well the clustering corresponds to ground truth labels. According to both metrics (Fig. 2C and Additional file 1: Fig. S1B), spCLUE generates the most reasonable spot embeddings and spatial clusters. Collectively, the results across all four metrics demonstrate the superior clustering performance of spCLUE in identifying spatial domains within the DLPFC dataset.

As an example, we visualized the clustering results of all methods alongside the spatial locations for slice 151672 (Fig. 2D). Clustering results for the other 11 slices are provided in Additional file 1: Figs. S2–S12. On slice 151672, spCLUE achieved the highest ARI score of 0.77, outperforming the next best methods, CCST and STAGATE, which both achieved an ARI of 0.62. Notably, spCLUE and CCST were the only methods capable of accurately identifying the entirety of layer 3 in this slice. We also examined the spot embeddings generated by the seven representation-learning methods, including spCLUE, for this slice (Fig. 2E). These embeddings were used to construct PAGA graphs [38], which were evaluated for their ability to preserve the topological structure of the cortex data. The PAGA graph derived from spCLUE’s embeddings demonstrated a well-defined laminar organization, with stronger connectivity (thicker edges) within cortical layers compared to the connectivity between the white matter and cortical layers.

### spCLUE effectively identifies spatial domains in single-slice data across diverse SRT technologies

**Fig. 3:**
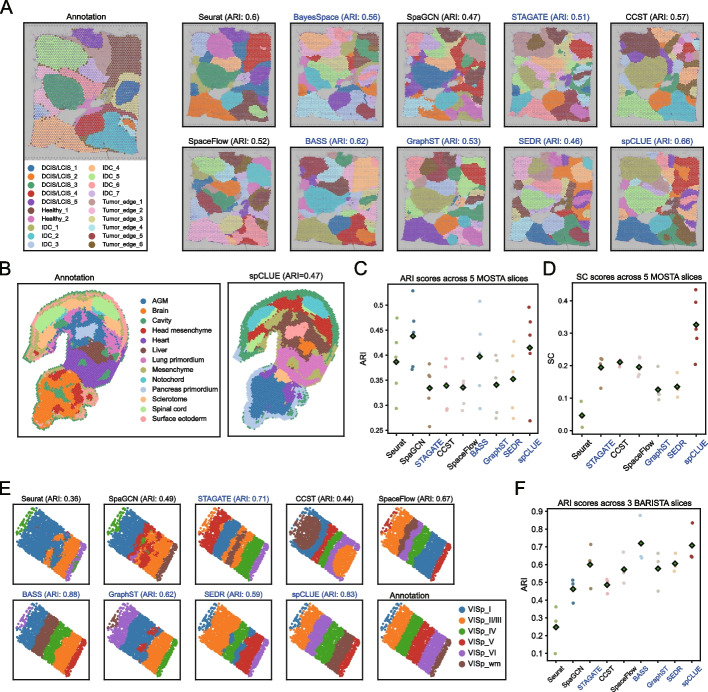
Spatial domain identification results on the BRCA, MOSTA, and BARISTA datasets. **A** Annotated and predicted spatial domains for the BRCA dataset. DCIS: ductal carcinoma in situ; LCIS: lobular carcinoma in situ; Healthy: healthy tissue; IDC: invasive ductal carcinoma. **B** Annotated regions and predicted spatial domains by spCLUE on the MOSTA dataset (slice E2S2). **C** ARI scores on the five MOSTA slices. The circles denote ARI scores on individual slices, and the diamonds mark the average ARI score across the slices. The methods are ordered based on publication year, and methods applicable to multi-slice data are highlighted with the blue color. **D** SC scores on the five MOSTA slices. **E** Annotated and predicted spatial domains for the BARISTA dataset (slice 2). **F** ARI scores on the three BARISTA slices

We further evaluated the performance of spCLUE on five SRT datasets generated using different technologies. Given the growing utility of SRT in studying tumor microenvironments, we first assessed spCLUE on a human breast cancer (BRCA) dataset sequenced with the 10x Visium technology [39]. This dataset includes 3798 spots and 36,601 genes, with annotations for 20 regions based on H&E staining [25]. When applied to this single-slice dataset, spCLUE achieved the highest ARI score of 0.66 and the highest NMI value of 0.72 among all compared methods (Fig. 3A and Additional file 1: Fig. S13). For invasive ductal carcinoma (IDC) regions such as IDC_5 and IDC_7, spCLUE was the only method to accurately cluster these regions, whereas alternative methods split them into multiple smaller clusters (Additional file 1: Fig. S14).

Second, we evaluated spCLUE on a MOSTA dataset containing mouse embryo data at the E9.5 developmental stage, generated using the Stereo-seq technology [6]. This dataset consists of five slices (E1S1, E2S1, E2S2, E2S3, E2S4), with 4356 to 5913 spots and 23,398 to 25,568 genes in each slice (Fig. 3B). The spots in each slice were annotated with 12 to 14 tissue labels. We compared the performance of spCLUE with eight alternative methods excluding BayesSpace, which is tailored specifically for SRT data from the 10x Genomics platform (Additional file 1: Fig. S15). Based on the average ARI (Fig. 3C) and NMI (Additional file 1: Fig. S16) scores, spCLUE outperformed seven of the alternative methods, with only slightly lower scores than SpaGCN. Additionally, spCLUE demonstrated the best clustering performance based on average SC (Fig. 3D) and CH scores (Additional file 1: Fig. S16), outperforming all other methods in these two metrics.

Third, we evaluated spCLUE on a dataset sequenced by the BaristaSeq technology, which we refer to as the BARISTA dataset [7, 40]. It consists of three slices of mouse primary visual cortex samples, each with 1525 to 2042 spots and 79 genes. The spots in these slices have been annotated with six layers. We applied spCLUE and eight alternative methods (excluding BayesSpace) to individual slices (Fig. 3E and Additional file 1: Figs. S17–S18). On all three slices, we observed a good correspondence between the annotated tissue layers and the inferred domains by spCLUE. Additionally, spCLUE and BASS were the only two methods to achieve average ARI scores exceeding 0.7, while the third-best method, SEDR, attained an average ARI of 0.61 (Fig. 3F).

**Fig. 4:**
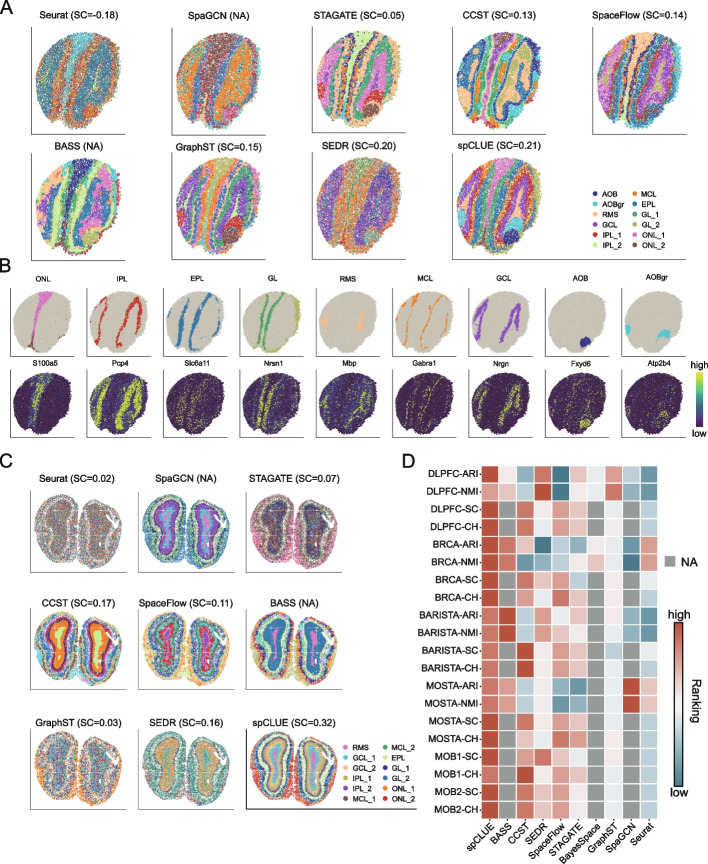
Spatial domain identification results on MOB1 and MOB2 datasets. **A** Inferred spatial domains for the MOB1 dataset. In spCLUE’s results, the inferred domains were annotated using the Allen Brain Reference Atlas [41] as a reference. AOB: accessory olfactory bulb; AOBgr: AOB granular layer; RMS: rostral migratory stream; GCL: granule cell layer; IPL: internal plexiform layer; MCL: mitral cell layer; EPL: external plexiform layer; GL: glomerular layer; ONL: olfactory nerve layer. **B** Known marker genes of spatial domains identified by spCLUE on the MOB1 dataset. **C** Inferred spatial domains for the MOB2 dataset. In spCLUE’s results, the inferred domains were annotated using labels in Xu et al. [25] as a reference. **D** Method comparison across six single-slice datasets. For each dataset and applicable metric (ARI, NMI, SC, or CH), we ranked the methods with a rank of 1 indicating the best performance. For methods that do not generate low-dimensional spot embeddings, SC and CH rankings are displayed as NAs. Methods are ordered based on their average rankings

Lastly, we evaluated the performance of spCLUE on two datasets of mouse olfactory bulbs. The first dataset (MOB1) [5], consisting of a single slice, was generated using the Slide-seqV2 technology. We compared spCLUE with eight benchmark methods (excluding BayesSpace) based on SC and CH scores since spot-level annotation for this dataset was not available. Among the methods, spCLUE achieved the highest SC score and the second-highest CH score (Additional file 1: Fig. S19). To better interpret spCLUE’s inferred spatial domains, we aligned them with known anatomical layers using the annotation of coronal mouse brain sections from the Allen Reference Atlas as a reference (Fig. 4A) [41]. spCLUE successfully identified distinct regions corresponding to the AOB, AOBgr, and RMS layers, which many other methods failed to distinguish. Furthermore, we identified differentially expressed genes (DEGs) between these domains (Additional file 1: Fig. S20; Additional file 1: Supplementary Methods), many of which overlapped with known marker genes (Fig. 4B). For example, *S100a5* for ONL [42], *Slc6a11* for EPL [43], *Nrsn1* for GL [1], *Mbp* for RMS [44], *Fxyd6* for AOB [20], and *Atp2b4* for AOBgr [20]. Additionally, *Pcp4*, identified as an upregulated gene in both IPL and GCL, demonstrated shared expression between these two adjacent layers, consistent with prior findings [1, 45]. The second dataset (MOB2), also consisting of a single slice, was sequenced using the Stereo-seq technology. On this dataset, spCLUE achieved the highest SC and CH scores (Fig. 4C and Additional file 1: Fig. S21), highlighting its superior clustering performance. Moreover, spCLUE detected well-defined spatial structures consistent with known marker genes (Additional file 1: Figs. S22–S23), further demonstrating its ability to accurately delineate biological layers.

To provide a comprehensive comparison of spatial domain identification methods across the six datasets (DLPFC, BRCA, BARISTA, MOSTA, MOB1, and MOB2), we ranked the methods based on their average performance for each of the four evaluation metrics (ARI, NMI, SC, and CH) across single slices within each dataset, with a rank of 1 representing the best performance. Among the methods, spCLUE achieved the highest overall ranking, followed by BASS, CCST, and SEDR (Fig. 4D). These consistent results highlight spCLUE’s robust ability to identify spatial domains effectively and its suitability for datasets generated using diverse SRT technologies.

### spCLUE identifies spatial domains by integrating multi-slice data

**Fig. 5:**
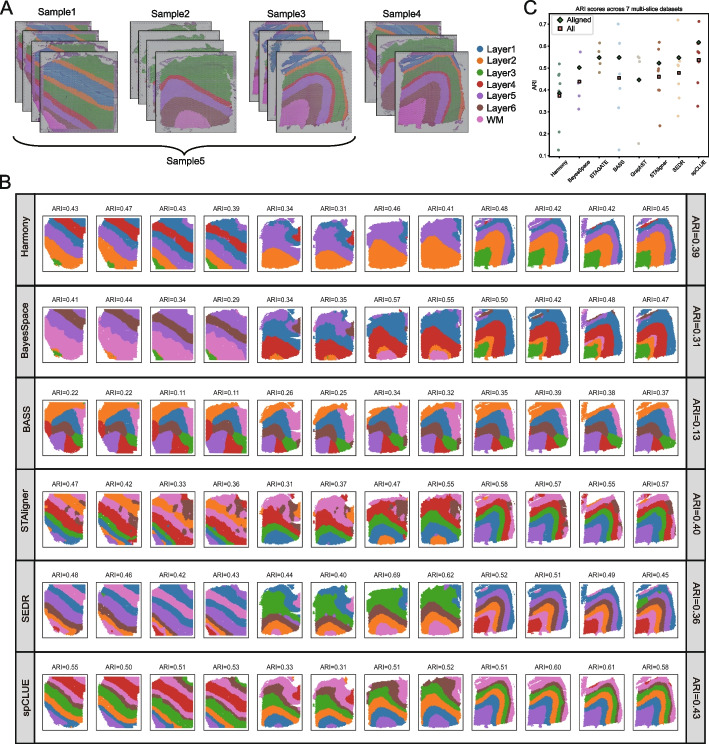
Spatial domain identification results on multi-slice datasets. **A** Five multi-slice samples based on the DLPFC dataset. **B** Spatial domain identification results on sample 5, by integrating all 12 DLPFC slices. **C** ARI scores were calculated for the seven multi-slice samples, which include five samples from the DLPFC dataset (samples 1–5), as well as one sample each from the MOSTA and BARISTA datasets. The diamonds mark the average ARI scores across spatially aligned data (samples 1–3 of DLPFC data and BARISTA data), and the squares mark the average ARI scores across all (aligned and unaligned) data

For spatial transcriptomics data consisting of multiple slices from the same organ or tissue type, integrative analysis is crucial for deriving coherent spatial domains that are directly comparable across slices. Such an approach facilitates a holistic understanding of gene expression patterns and biological functions. To evaluate spCLUE’s performance on multi-slice data, we first applied it to the DLPFC dataset, which includes 12 tissue slices from three samples. Each sample contains four adjacent slices: sample 1 (slices 151507, 151508, 151509, 151510), sample 2 (slices 151669, 151670, 151671, 151672), and sample 3 (slices 151673, 151674, 151675, 151676) (Fig. 5A). When applying spCLUE to these samples, each slice was treated as a distinct batch to account for potential technical effects between slices. Furthermore, to evaluate spCLUE’s ability to integrate unaligned slices from different subjects, we constructed two additional samples: sample 4, comprising one slice from each of the three samples (slices 151507, 151672, and 151673), and sample 5, containing all 12 slices.

We compared the integration performance of spCLUE with seven other methods applicable to multi-slice data (Additional file 1: Figs. S24–S27). STAGATE and GraphST were excluded from analyses of sample 4 and sample 5, as they are only suitable for spatially aligned slices. spCLUE achieved the highest ARI scores on samples 1, 2, and 5, as well as the highest SC and CH scores across all five samples (Additional file 1: Fig. S28). For instance, we visualized the integration results for sample 5, which includes 12 slices (Fig. 5B). The layer structures identified by spCLUE aligned well with the annotated cortical layers, resulting in the highest ARI score, followed by STAligner and Harmony. Importantly, spCLUE successfully identified the white matter layer across all slices and subjects, a task that proved challenging for most other methods. While the non-spatial integration method Harmony achieved the third-highest ARI score, its inferred clusters for the second subject primarily distinguished between two layer sections, offering limited biological insights for practical applications.

We then evaluated the multi-slice integration performance on the MOSTA and BARISTA datasets, comprising five and three slices, respectively. BayesSpace was excluded from both datasets due to technological incompatibility, while STAGATE and GraphST were excluded from the MOSTA dataset because it contains unaligned slices. spCLUE ranked among the top two methods for both datasets. On the MOSTA dataset, spCLUE achieved an ARI score of 0.33, followed by BASS, which scored 0.32 (Additional file 1: Fig. S29). For the BARISTA dataset, spCLUE attained an ARI score of 0.71, closely following SEDR, which had a score of 0.72 (Additional file 1: Fig. S30).

**Fig. 6:**
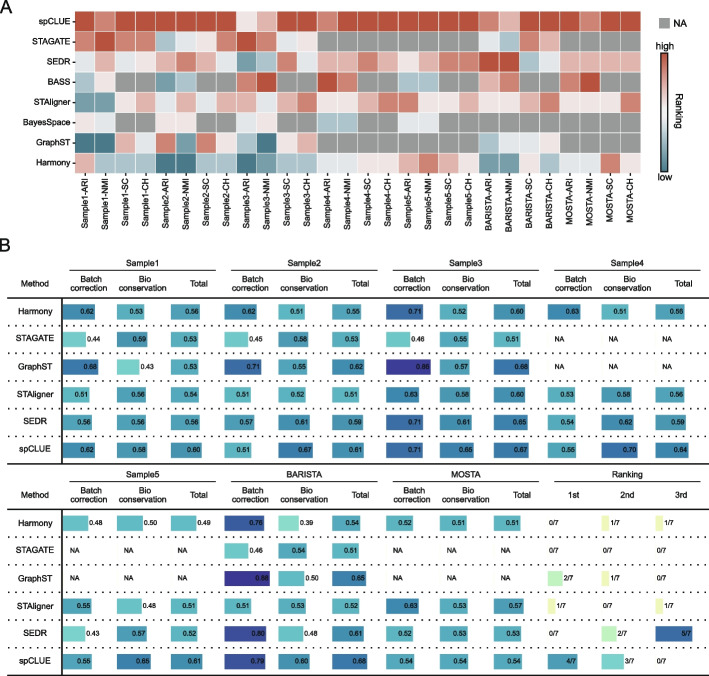
Method comparison on multi-slice datasets. **A** For each dataset, we ranked the methods based on four evaluation metrics (ARI, NMI, SC, or CH), with a rank of 1 indicating the best performance. For methods that do not generate low-dimensional spot embeddings, SC and CH rankings are displayed as NAs. Methods are ordered based on their average rankings. **B** Bio-conservation and batch-correction scores calculated using the scIB method. Both metrics range from 0 to 1, with 1 representing the best performance. The total score was computed as the average of the bio-conservation and batch-correction scores. The last three ranking columns show the frequency with which each method ranked among the top three positions across the seven datasets

To provide an overall comparison, we evaluated the eight methods across all multi-slice datasets. Based on clustering accuracy across the datasets (Fig. 5C) and performance metrics for individual slices (Additional file 1: Fig. S28), spCLUE demonstrated the highest overall accuracy among the methods. Additionally, we ranked the eight methods for each multi-slice dataset using the four evaluation metrics (ARI, NMI, SC, and CH), with a rank of 1 indicating the best performance. spCLUE consistently ranked at or near the top for most datasets and metrics, achieving the best average ranking among the methods (Fig. 6A).

To further assess spCLUE’s effectiveness in mitigating batch effects between slices and subjects for the integration of multi-slice spatial transcriptomics data, we compared it against five other methods capable of embedding spots from different slices into a shared latent space. This evaluation used the scIB method [46], which calculates two summary scores for each method on each dataset. The bio-conservation score measures a method’s ability to preserve biological variation from the original data, combining metrics such as ARI, NMI, and the average silhouette width relative to annotated layers. The batch-correction score quantifies a method’s ability to remove batch effects from spot embeddings, incorporating metrics such as the graph integration local inverse Simpson’s index [29] and the average silhouette width relative to batches. Based on the total score (the average of the bio-conservation and batch-correction scores), spCLUE consistently outperformed other methods, ranking first on four of the seven datasets and second on the remaining three. This demonstrates spCLUE’s strong ability to balance the preservation of biological variation with the removal of batch effects across diverse multi-slice datasets (Fig. 6B).

As the ground truth batch effects in real datasets are unknown, we conducted an additional simulation study to further evaluate the performance of different methods in removing batch effects on spatial transcriptomics data with controlled batch variations. Specifically, we simulated three datasets using the BARISTA, DLPFC (sample 3), and MOSTA datasets as references (Additional file 1: Supplementary Methods). We compared spCLUE with the five multi-slice methods that are able to learn spot representations using the three simulated datasets (Additional file 1: Fig. S31). In all cases, direct dimensionality reduction without batch effect correction failed to produce biologically meaningful clusters, as the clustering results were dominated by batch effects. Applying multi-slice integration methods alleviated this issue to varying degrees. On the BARISTAsim dataset, spCLUE achieved the highest ARI score of 0.98. The identified spatial domains showed near-perfect correspondence with the true labels and were no longer confounded by batch effects. In contrast, embeddings learned by Harmony, STAGATE, GraphST, and SEDR still exhibited strong batch-associated patterns. spCLUE also achieved the highest ARI scores on the DLPFCsim and MOSTAsim datasets. We further evaluated the methods using bio-conservation and batch-correction scores computed by the scIB package (Additional file 1: Fig. S31D). spCLUE consistently achieved the best overall scores across all three datasets, highlighting its unique ability to preserve biologically meaningful structures while effectively removing confounding batch effects.

To evaluate the scalability of spCLUE, we performed an additional analysis to compare the computational time and maximum memory usage of spCLUE and alternative methods on both single-slice and multi-slice datasets (Additional file 1: Figs. S32–S33). For single-slice analysis, we compared spCLUE with nine alternative methods. spCLUE was among the fastest methods overall, becoming only slightly slower than SEDR on larger datasets such as MOB1 and MOB2. In terms of memory usage, spCLUE was also among the four most efficient methods across datasets. For multi-slice integration, we compared spCLUE with seven alternative methods. spCLUE was the third fastest method. It was slightly slower than Harmony and SEDR, but required less memory than both of these methods.

### Graph contrastive learning contributes to spCLUE’s performance

**Fig. 7:**
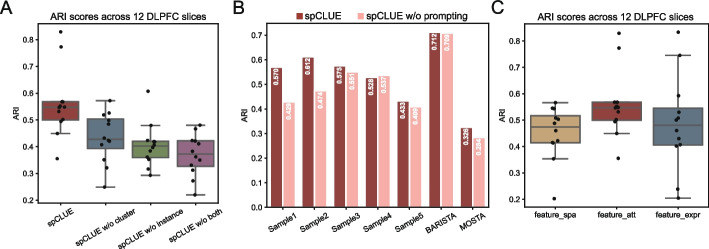
Ablation study of the spCLUE model. **A** ARI scores of the full spCLUE model and three ablated versions on individual slices in the DLPFC dataset. **B** Overall ARI scores of spCLUE and “spCLUE w/o prompting” on seven multi-slice datasets. **C** Distribution of ARI scores based on spatial domains inferred from three types of spot embeddings, feature_spa ($\varvec{\textrm{Z}}_{spa}$), feature_att ($\varvec{\textrm{Z}}$; used in spCLUE), and feature_expr ($\varvec{\textrm{Z}}_{expr}$), on the DLPFC dataset

spCLUE learns spot representations from spatial transcriptomics data using the idea of graph contrastive learning, by combining an instance contrastive module and a clustering contrastive module (see “Methods” for details). To evaluate the contributions of these components, we conducted an ablation study by removing each module individually, creating two ablated versions of spCLUE: “spCLUE w/o instance” (without the instance contrastive module) and “spCLUE w/o cluster” (without the clustering contrastive module). Additionally, we tested a backbone-only network version, referred to as “spCLUE w/o both,” which relies solely on reconstructing expression profiles from the auto-encoder without either contrastive module. Using the DLPFC dataset as an example, we illustrate the ablation results for the single-slice version of spCLUE (Fig. 7A). The full version of spCLUE achieved the highest median ARI score, outperforming all three ablated versions. Among the ablated models, “spCLUE w/o cluster” performed similar as “spCLUE w/o instance” while the backbone-only network, “spCLUE w/o both,” ranked last. The complete ablation results for all datasets are summarized in Additional file 1: Table S3, which confirm that the full spCLUE model consistently outperforms its ablated versions. These results highlight the critical role of integrating the instance contrastive module and the clustering contrastive module, as their synergy enhances the representation of spatial domain patterns in the embedding space, enabling the model to achieve superior performance in spatial domain identification.

To assess the contribution of the batch prompting module to the integration performance of spCLUE in multi-slice analysis, we created an ablated version by removing the batch information prompting module, referred to as “spCLUE w/o prompting.” A comparison between spCLUE and “spCLUE w/o prompting” across seven multi-slice datasets (samples 1–5 of DLPFC, BARISTA, and MOSTA) revealed that spCLUE outperformed the ablated version in clustering accuracy on five of the datasets. The two methods exhibited similar performance on the remaining two datasets (Fig. 7B). These results indicate that incorporating the batch prompting module generally enhances the accuracy of spatial domain identification. Furthermore, we compared the spot embeddings derived from the spatial graph ($\varvec{\textrm{Z}}_{spa}$), the expression graph ($\varvec{\textrm{Z}}_{expr}$), and the unified spot embeddings ($\varvec{\textrm{Z}}$) to evaluate the role of the attention module. The results demonstrate that the unified spot embeddings, generated by the attention mechanism, outperformed the single-view embeddings based on either spatial or gene expression information alone (Fig. 7C).

To further understand how data characteristics might affect the performance of spCLUE, we analyzed the relationship between spCLUE’s accuracy and two statistics for each dataset, label smoothness (LS) and label entropy (LE) (Additional file 1: Supplementary Methods). LS describes how similar the annotated spatial domains are between neighboring spots in a dataset. The value of LS ranges from 0 to 1, with higher values indicating more consistent labeling across nearby spots. LE measures how evenly the labels are distributed across the tissue. This measure helps assess whether the annotated spatial domains are balanced or skewed toward a few dominant labels, with higher values indicating more diversity in the label distribution. We observed that spCLUE tended to achieve higher accuracy on datasets with larger LS (where annotated spatial domains are more consistent across neighboring spots) and smaller LE (where the distribution of annotated domains is less diverse) (Additional file 1: Fig. S34).

## Discussion

Spatial domain identification is a critical task in the analysis of spatially resolved transcriptomics data. In this study, we presented spCLUE, a novel spatial domain identification method based on a graph contrastive learning framework designed to analyze both single-slice and multi-slice SRT datasets. The spCLUE framework includes four main modules: (1) a backbone module for reconstructing gene expression profiles, (2) an instance contrastive learning module for generating consistent spot representations, (3) a clustering contrastive module to amplify clustering signals in the embedding space, and (4) an attention module for integrating spot representations derived from spatial locations and gene expression profiles. For multi-slice analysis, spCLUE introduces a batch information prompting module, which enables the simultaneous integration of multiple samples by guiding the network to correct for batch effects in the embedding space. The effectiveness of these tailored designs was confirmed through comprehensive ablation studies, demonstrating their contributions to spCLUE’s robust performance.

We evaluated spCLUE’s performance across multiple tasks, including single-slice and multi-slice analyses. For single-slice analysis, spCLUE was compared against nine other methods across six datasets generated by diverse technologies, including 10x Visium, Slide-SeqV2, Stereo-seq, and BaristaSeq. Among these methods, spCLUE achieved the best overall performance and ranking, demonstrating its ability to identify spatial domains effectively across various experimental platforms. For multi-slice analysis, spCLUE demonstrated leading performance among eight methods across three datasets and seven samples, showing its capability to integrate spatial transcriptomics data from multiple slices. Beyond standard metrics such as ARI, NMI, SC, and CH, spCLUE’s integration performance was further validated using the bio-conservation and batch-correction scores from the scIB [46] package. These results highlight spCLUE’s robust ability to preserve biological variation and mitigate batch effects while integrating slices from different subjects or developmental stages.

In this work, we focused on spatial domain identification using only gene expression and spatial location information from SRT data. In some spatial transcriptomics studies, additional data modalities, such as histological images, are available alongside spatial and expression information. Methods like SpaGCN [17], DeepST [47], and stMMR [48] incorporate histological features as an additional layer of input to enhance spatial domain identification. Similarly, methods like IRIS [49] leverage annotated single-cell RNA sequencing data from the same tissue type to guide the analysis of SRT data. On the one hand, integrating such auxiliary information can potentially improve spatial domain identification, particularly in datasets where gene expression is sparse. On the other hand, relying on external data may limit the applicability of these methods in studies where such resources are unavailable. To balance flexibility and performance, a future direction for spCLUE is to incorporate additional data modalities as optional inputs. For example, when histology images are available, spCLUE could leverage them as a third view within the multi-view graph construction framework. Moreover, advances in sequencing technologies, such as DBiT-seq [50] and spatial-CITE-seq [51], have enabled multi-omics profiling while preserving spatial resolution. With spCLUE’s modular architecture designed for processing multi-view graphs, it is well-suited to be extended to multi-omics data. One potential approach would involve constructing modality-specific expression graphs and refining them using spatial information to improve representation learning. In summary, we have demonstrated that spCLUE is a novel and powerful tool for clustering diverse SRT datasets and integrating data from different subjects. As the availability of SRT datasets continues to grow, we anticipate that spCLUE will become a useful tool for deriving biologically meaningful domains in spatial transcriptomics analysis.

## Conclusions

In this article, we introduce spCLUE, a novel approach for unified analysis of spatial transcriptomics data across both single-slice and multi-slice settings. Built on a contrastive learning framework, spCLUE learns informative spot-level representations and enables effective integration of multi-slice data from both aligned and unaligned samples. Through comprehensive experiments on diverse datasets, we demonstrate spCLUE’s ability to uncover biologically meaningful spatial domains across a variety of tissues and conditions. In summary, spCLUE offers a powerful and flexible solution for identifying spatial domains and integrating complex spatial transcriptomics data.

## Methods

### Data preprocessing

We will first introduce detailed components of spCLUE in the special case with a single slice ($L=1$), and then introduce how the model is extended for multi-slice data ($L>1$). We follow standard preprocessing steps for single-cell transcriptomics data and these steps are implemented by the Scanpy package [52]. For gene expression data, we first filter out genes that are not expressed in any spot and obtain the filtered count matrix $\textbf{Y} = [y_{ij}]_{m\times n_0}$. Then, we perform library-size normalization and log-transformation to obtain $\widehat{\textbf{Y}} = [\hat{y}_{ij}]_{m\times n_0}$, where

2 $$\begin{aligned} \hat{y}_{ij} = \textrm{log} \left( \frac{10^{4}}{\sum _{j=1}^{n_0}y_{ij}} y_{ij} + 1\right) , i=1,2,\dots , m,\ j=1,2,\dots , n_0. \end{aligned}$$

Next, we perform standardization on the gene expression levels to obtain $\widetilde{\textbf{Y}} = [\widetilde{y}_{ij}]_{m\times n_0}$, where

3 $$\begin{aligned} \widetilde{y}_{ij} = \frac{\widehat{y}_{ij} - \mu _{j}}{\sigma _{j}},\quad \mu _{j} = \frac{1}{m}\sum \limits _{i = 1}^m \hat{y}_{ij}, \quad \sigma _{j} = \sqrt{\frac{1}{m - 1}\sum \limits _{i = 1}^m \left( \hat{y}_{ij} - \mu _{j}\right) ^2}. \end{aligned}$$

Finally, we perform the principal component analysis (PCA) on $\widetilde{\textbf{Y}}$ and denote the transformed values on the first *n* principal components as $\varvec{\textrm{X}}\in \mathbb {R}^{m\times n}$, which is used as the input to spCLUE.

### Multi-view graph construction

To simultaneously incorporate spatial and gene expression information, we construct a spatial neighbor graph and an expression neighbor graph using spot locations and gene expression profiles, respectively. The *K*-nearest neighbors (KNN) algorithm [53] is applied to the spot location matrix $\textbf{S} = [s_{ij}]_{m \times 2}$ to build the spatial neighbor graph based on Euclidean distance. The adjacency matrix of the spatial neighbor graph, $\varvec{\textrm{A}}_{spa} = \left[a^{spa}_{ij}\right]_{m \times m}$, is defined as:

4 $$\begin{aligned} a^{spa}_{ij} = \left\{ \begin{array}{ll} 1, & j\in \mathcal {N}_{spa}(i) \\ 0, & j\notin \mathcal {N}_{spa}(i) \end{array} \right. , \end{aligned}$$

where $\mathcal {N}_{spa}(i)$ represents the index set of spatial nearest neighbors of spot *i*. As for the expression neighbor graph, the nearest neighbors are searched based on the Pearson correlation coefficient. Then, the expression adjacency matrix, $\varvec{\textrm{A}}_{expr} = \left[a^{expr}_{ij}\right]_{m \times m}$, is defined as

5 $$\begin{aligned} a^{expr}_{ij} = \left\{ \begin{array}{ll} 1, & j\in \mathcal {N}_{expr}(i) \\ 0, & j\notin \mathcal {N}_{expr}(i) \end{array} \right. , \end{aligned}$$

where $\mathcal {N}_{expr}(i)$ represents the index set of expression nearest neighbors of spot *i*.

After constructing the adjacency matrices, we construct the following hybrid adjacency matrices [18] to incorporate self-connections:

6 $$\begin{aligned} \widehat{\varvec{\textrm{A}}}_{*} = \lambda \textbf{I} + (1 - \lambda ) \varvec{\textrm{A}}_{*}, \quad \varvec{\textrm{A}}_{*} \in \{\varvec{\textrm{A}}_{spa}, \varvec{\textrm{A}}_{expr}\}, \end{aligned}$$

where $\textbf{I}$ is the identity matrix. The hyperparameter $\lambda$ controls the weight of self-connections, and we set $\lambda = 0.3$ in accordance with prior work [22]. Finally, we normalize each adjacency matrix by the degrees of the nodes (spots):

7 $$\begin{aligned} \widetilde{\varvec{\textrm{A}}}_{*} = \textbf{D}_{*}^{-1/2} \widehat{\varvec{\textrm{A}}}_{*} \textbf{D}_{*}^{-1/2}, \quad \widehat{\varvec{\textrm{A}}}_{*} \in \{\widehat{\varvec{\textrm{A}}}_{spa}, \widehat{\varvec{\textrm{A}}}_{expr}\}, \end{aligned}$$

where $\textbf{D}_{*} \in \mathbb {R}^{m\times m}$ is the degree matrix of $\widehat{\varvec{\textrm{A}}}_{*}$, and $d^{*}_{ii} = \sum _{j=1}^m \widehat{a}_{ij}^{*}$ is the degree of spot *i*. These symmetric normalized adjacency matrices are then input to the graph contrastive learning module to extract spot representations.

### Graph contrastive learning

The graph contrastive learning module in spCLUE adopts an encoder-decoder framework to train the network, with the encoder based on a GCN architecture. Before inputting data into the GCN, moderate random noise is added to the expression matrix $\varvec{\textrm{X}} = [x_{ij}]_{m \times n}$ to produce a perturbed matrix $\widetilde{\textbf{X}} = [\widetilde{x}_{ij}]_{m \times n} \in \mathbb {R}^{m \times n}$:

8 $$\begin{aligned} \widetilde{x}_{ij} = b_{ij} \cdot (x_{ij} + \alpha \cdot \varepsilon _{ij}), i = 1, 2, \ldots , m, \; j = 1, 2, \ldots , n, \end{aligned}$$

where $\varepsilon _{ij} \sim \mathcal {N}(0, 1)$ represents Gaussian noise, and $\alpha$ controls the intensity of the noise. Additionally, $b_{ij} \sim \mathcal {B}(p)$ is a masking flag following a Bernoulli distribution with masking probability *p*. For each graph $\mathscr {G}_{*} = (\widetilde{\varvec{\textrm{A}}}_{*}, \widetilde{\textbf{X}}), \widetilde{\varvec{\textrm{A}}}_{*} \in \{\widetilde{\varvec{\textrm{A}}}_{spa}, \widetilde{\varvec{\textrm{A}}}_{expr}\}$, spCLUE applies edge masking before inputting the graph into the GCN encoder:

9 $$\begin{aligned} \mathcal {C}(\mathbf {\widetilde{\varvec{\textrm{A}}}_{*}}) = \mathbf {\widetilde{\varvec{\textrm{A}}}_{*}} \odot \textbf{M}, \end{aligned}$$

where $\mathcal {C}(\cdot )$ is the graph corruption function, $\textbf{M} = [m_{ij}]_{m\times m}$ is a masking matrix with $m_{ij}\sim \mathcal {B}(p_m)$, and $\odot$ represents the Hadamard product. These augmentation steps collectively enhance the robustness of the training process.

For each graph, we use a two-layer GCN to obtain the spot representations $\varvec{\textrm{Z}}_{*}\in \mathbb {R}^{m\times d}$:

10 $$\begin{aligned} \varvec{\textrm{Z}}_{*} = \textrm{GCN}(\widetilde{\varvec{\textrm{A}}}_{*}, \widetilde{\textbf{X}}) = \sigma \left( \mathcal {C}\left( \widetilde{\varvec{\textrm{A}}}_{*}\right) \left( \sigma \left( \mathcal {C}\left( \widetilde{\varvec{\textrm{A}}}_{*}\right) \widetilde{\textbf{X}} \textbf{W}_{11} \right) \right) \textbf{W}_{12} \right) , \end{aligned}$$

where $\textbf{W}_{11} \in \mathbb {R}^{n\times d_1}$ and $\textbf{W}_{12}\in \mathbb {R}^{d_1\times d}$ are parameters of the GCN. $\sigma (\cdot )$ represents the ELU activation function.

To encourage similarity between representations learned from the two graphs and enhance clustering signals in the embedding spaces, we employ an instance contrastive module and a clustering contrastive module, each tailored to achieve these respective goals. Drawing inspiration from the SimCLR method [54], both modules use a projection head to map the representations into a deeper feature space, where contrastive learning is applied. Each projection head consists of a two-layer multilayer perceptron with ReLU activation:

11 $$\begin{aligned} \begin{array}{l} \varvec{\textrm{H}}_{*} = \textrm{ReLU}\left( \varvec{\textrm{Z}}_{*}\textbf{W}_{21}\right) \textbf{W}_{22}, \quad \varvec{\textrm{H}}_{*}\in \left\{ \varvec{\textrm{H}}_{spa}, \varvec{\textrm{H}}_{expr}\right\} ; \\ \varvec{\textrm{Q}}_{*} = \textrm{softmax}\left( \textrm{ReLU}\left( \varvec{\textrm{Z}}_{*}\textbf{W}_{31}\right) \textbf{W}_{32}\right) ,\quad \varvec{\textrm{Q}}_{*}\in \left\{ \varvec{\textrm{Q}}_{spa}, \varvec{\textrm{Q}}_{expr}\right\} , \end{array} \end{aligned}$$

where $\textbf{W}_{21}\in \mathbb {R}^{d\times d_2}, \textbf{W}_{22}\in \mathbb {R}^{d_2\times f}, \textbf{W}_{31}\in \mathbb {R}^{d\times d_2},\ \text {and}\ \textbf{W}_{32}\in \mathbb {R}^{d_2\times N_c}$ are parameters of spCLUE.

For the instance contrastive module, we align representations under the two views using an instance loss $\mathcal {L}_{ins}$, which is calculated as:

12 $$\begin{aligned} \mathcal {L}_{ins} = -\frac{1}{m}\sum \limits _{i=1}^m\textrm{log}\left( \frac{\exp \left(s\left(\varvec{\textrm{h}}_i^{spa},\varvec{\textrm{h}}_i^{expr}\right)/\tau _{ins}\right)}{\sum _{k=1}^m \exp \left(s\left(\varvec{\textrm{h}}_i^{spa},\varvec{\textrm{h}}_k^{expr}\right)/\tau _{ins}\right)}\right) -\frac{1}{m}\sum \limits _{i=1}^m\textrm{log}\left( \frac{\exp \left(s\left(\varvec{\textrm{h}}_i^{expr},\varvec{\textrm{h}}_i^{spa}\right)/\tau _{ins}\right)}{\sum _{k=1}^m \exp \left(s\left(\varvec{\textrm{h}}_i^{expr},\varvec{\textrm{h}}_k^{spa}\right)/\tau _{ins}\right)}\right) , \end{aligned}$$

where $\varvec{\textrm{h}}_i^{*}$ denotes the *i-*th row of $\textbf{H}_{*}$ and $s(\cdot , \cdot )$ denotes the cosine similarity. $\tau _{ins}$ is a temperature hyperparameter to control the smoothness of the contrastive loss. This module encourages alignment between the representations of the same spot between the spatial neighbor graph and the expression neighbor graph. This helps ensure that the learned embeddings from the two data modalities are consistent at the individual spot level, which improves the robustness of the learned representation and promotes the fusion of complementary information from both graphs. Notably, different from the common formulation of instance contrastive loss [54], features of different spots on the same graph are not considered in the denominator to minimize their similarity, as the enforced dissimilarity constraint is opposite to the smoothing role of GCN encoder. For the clustering contrastive module, $\varvec{\textrm{Q}}_{*}\in \left\{ \varvec{\textrm{Q}}_{spa}, \varvec{\textrm{Q}}_{expr}\right\}$ captures cluster membership information, and we optimize the consistency of the two sets of cluster membership using a clustering contrastive loss $\mathcal {L}_{cls}=\mathcal {L}_{cls}^{spa} + \mathcal {L}_{cls}^{expr}$, where

13 $$\begin{aligned} \begin{array}{l} \mathcal {L}_{cls}^{spa} = -\frac{1}{N_c}\sum \limits _{j=1}^{N_c}\textrm{log}\left( \frac{\exp \left(s\left(\varvec{q}_j^{spa},\varvec{q}_j^{expr}\right)/\tau _{cls}\right)}{ \sum _{k=1}^{N_c} \exp \left(s\left(\varvec{q}_j^{spa},\varvec{q}_k^{expr}\right)/\tau _{cls}\right) +\sum _{k\ne j}\exp \left(s\left(\varvec{q}_j^{spa},\varvec{q}_k^{spa}\right)/\tau _{cls}\right)}\right) + \mathcal {L}_{reg}^{spa},\\ \mathcal {L}_{cls}^{expr} = -\frac{1}{N_c}\sum \limits _{j=1}^{N_c}\textrm{log}\left( \frac{\exp \left(s\left(\varvec{q}_j^{expr},\varvec{q}_j^{spa}\right)/\tau _{cls}\right)}{\sum _{k=1}^{N_c} \exp \left(s\left(\varvec{q}_j^{expr},\varvec{q}_k^{spa}\right)/\tau _{cls}\right) +\sum _{k\ne j} \exp \left(s\left(\varvec{q}_j^{expr},\varvec{q}_k^{expr}\right)/\tau _{cls}\right)}\right) + \mathcal {L}_{reg}^{expr}, \end{array} \end{aligned}$$

and $\varvec{q}_{j}^{*}$ denotes the *j*th column of $\textbf{Q}_{*}$. $\tau _{cls}$ is a temperature hyperparameter to control the smoothness of the clustering contrastive loss. The regularization term is defined as $\mathcal {L}_{reg}^{*}=\sum _{j=1}^{N_c} \pi _j^{*}\textrm{log}\pi _j^{*}$, $\mathcal {L}_{reg}^{*} \in \left\{\mathcal {L}_{reg}^{spa}, \mathcal {L}_{reg}^{expr}\right\}$, where $\pi _j^{*}= \sum _{i=1}^m q_{ij}^{*} / \sum _{j=1}^{N_c}\sum _{i=1}^{m} q_{ij}^{*}$ denotes the normalized probability of pseudo cluster *j*. This module is designed to enhance the clustering signals without relying on explicit clustering or known cluster labels during training. The loss function contrasts representations of different pseudo clusters across the multi-view graph. It encourages spot representations within the same pseudo cluster (but from different graphs) to be similar. Meanwhile, the regularization term $\mathcal {L}_{reg}^{*}$ is based on the entropy of cluster assignment probabilities and prevents the model from collapsing to trivial solutions, such as assigning all spots into a single cluster. This regularization term enhances the distinguishability of spot representations and improves the stability of the clustering signals. Overall, the cluster-level contrastive loss facilitates the emergence of more distinct and well-separated cluster structures in the learned embedding space.

In order to reconstruct the gene expression data from the decoder, we first learn the integrated spot representations, $\varvec{\textrm{Z}}$, which are obtained through an attention block. We calculate the attention scores as follows:

14 $$\begin{aligned} \textbf{Att}_{*} = \textrm{tanh}\left( \varvec{\textrm{Z}}_{*}\textbf{W}_{41}\right) \textbf{W}_{42}, \quad \textbf{Att}_{*} \in \{\textbf{Att}_{spa}, \textbf{Att}_{expr}\}, \end{aligned}$$

where $\textbf{W}_{41} \in \mathbb {R}^{d \times d_3}$ and $\textbf{W}_{42} \in \mathbb {R}^{d_3}$ are learnable parameters. The resulting attention scores $\textbf{Att}_{*} = \left[att_{i}^{*}\right]_{m\times 1}$ represent the importance of each spot. These attention scores are then normalized into weights, $\varvec{\alpha }_{spa} = \left[\alpha ^{spa}_{ij}\right]_{m \times d}$ and $\varvec{\alpha }_{expr} = \left[\alpha ^{expr}_{ij}\right]_{m \times d}$, using the softmax function:

15 $$\begin{aligned} \begin{array}{l} \alpha ^{spa}_{ij} = \frac{\exp \left( att^{spa}_i\right) }{\exp \left( att^{spa}_i\right) + \exp \left( att^{expr}_i\right) }, \\ \alpha ^{expr}_{ij} = \frac{\exp \left( att^{expr}_i\right) }{\exp \left( att^{spa}_i\right) + \exp \left( att^{expr}_i\right) }. \end{array} \end{aligned}$$

Then, the integrated representations $\varvec{\textrm{Z}}$ are obtained by combining $\varvec{\textrm{Z}}_{spa}$ and $\varvec{\textrm{Z}}_{expr}$ using the normalized attention scores:

16 $$\begin{aligned} \varvec{\textrm{Z}} = \varvec{\alpha }_{spa} \odot \varvec{\textrm{Z}}_{spa} + \varvec{\alpha }_{expr} \odot \varvec{\textrm{Z}}_{expr}. \end{aligned}$$

For the reconstruction task, we use a symmetric decoder architecture to reconstruct the expression feature matrix $\widehat{\textbf{X}}$ as

17 $$\begin{aligned} \widehat{\textbf{X}} = \textrm{ReLU}\left( \varvec{\textrm{Z}}\textbf{W}_{12}^{\textrm{T}}\right) \textbf{W}_{11}^{\textrm{T}}. \end{aligned}$$

The corresponding reconstruction loss $\mathcal {L}_{rec}$ is defined as

18 $$\begin{aligned} \mathcal {L}_{rec} = \frac{1}{mn}\sum \limits _{i=1}^{m} \sum \limits _{j=1}^{n} (x_{ij} - \widehat{x}_{ij})^2. \end{aligned}$$

Finally, the spCLUE network is trained using a weighted sum of three losses (reconstruction loss, cluster contrastive loss, and instance contrastive loss), defined as:

19 $$\begin{aligned} \mathcal {L} = \mathcal {L}_{rec} + \mathcal {L}_{cls} + \kappa \mathcal {L}_{ins} , \end{aligned}$$

where $\kappa$ is the weight on the instance contrastive loss.

### Multi-slice graph contrastive learning

In this section, we introduce how the spCLUE model is extended for the integration of multiple slices of spatial transcriptomics data. For a collection of *L* slices, spCLUE first processes each count matrix following the procedure introduced in “Data preprocessing” section and obtains a set of preprocessed expression matrices $\mathcal {Y} = \{\widetilde{\textbf{Y}}_1, \widetilde{\textbf{Y}}_2, \cdots , \widetilde{\textbf{Y}}_L\}\ (\widetilde{\textbf{Y}}_l\in \mathbb {R}^{m_l\times n_0}$, $l=1,2,\cdots ,L$), where $m_l$ denotes the number of spots in slice *l*. Next, spCLUE performs PCA on the concatenated expression matrix of the *L* preprocessed expression matrices to obtain the input features $\varvec{\textrm{X}}\in \mathbb {R}^{m\times n}$, where $m=\sum _{i=1}^L m_i$ denotes the total number of spots in the *L* slices. Then, spCLUE constructs adjacency matrices $\widetilde{\varvec{\textrm{A}}}_{spa}^{(l)}$ and $\widetilde{\varvec{\textrm{A}}}_{expr}^{(l)}$ for each $\widetilde{\textbf{Y}}_{l}$, as explained in “Multi-view graph construction” section. Lastly, spCLUE arranges these spatial (or expression) adjacency matrices in a block diagonal form to obtain the ultimate spatial (or expression) adjacency matrix $\widetilde{\varvec{\textrm{A}}}_{*} = \textrm{diag}\{\widetilde{\varvec{\textrm{A}}}_{*}^{(1)}, \widetilde{\varvec{\textrm{A}}}_{*}^{(2)}, \cdots , \widetilde{\varvec{\textrm{A}}}_{*}^{(L)}\}, \widetilde{\varvec{\textrm{A}}}_{*}\in \{\widetilde{\varvec{\textrm{A}}}_{spa}, \widetilde{\varvec{\textrm{A}}}_{expr}\}$.

In order to correct potential batch effects between different slices, spCLUE considers the batch information through two batch embedding matrices $\textbf{B}_1=[\varvec{b}^{(1)}_1, \varvec{b}^{(1)}_2, \cdots , \varvec{b}^{(1)}_L]^{\textrm{T}}\in \mathbb {R}^{L\times n}$ and $\textbf{B}_2=\left[\varvec{b}^{(2)}_1, \varvec{b}^{(2)}_2, \cdots , \varvec{b}^{(2)}_L\right]^{\textrm{T}}\in \mathbb {R}^{L\times d}$ [55]. Every time we feed $\varvec{\textrm{X}}$ into spCLUE, $\varvec{\textrm{X}}$ is first corrected by the batch prompting embeddings to obtain $\varvec{\textrm{X}}^{(b)}=\left[\varvec{\textrm{x}}^{(b)}_1, \varvec{\textrm{x}}^{(b)}_2, \cdots , \varvec{\textrm{x}}^{(b)}_m\right]^{\textrm{T}}\in \mathbb {R}^{m\times n}$:

20 $$\begin{aligned} \varvec{\textrm{x}}_i^{(b)} = \varvec{\textrm{x}}_i - \varvec{b}_{l_i}^{(1)}, i=1, 2, \cdots , m, \end{aligned}$$

where $l_i$ is the batch (slice) id of spot *i*. Under the guidance of the batch embeddings, $\varvec{\textrm{X}}^{(b)}$ is used to construct the multi-view graph for contrastive graph learning, which outputs aligned embeddings $\varvec{\textrm{Z}}$ in the latent space. Then, for the reconstruction of the expression matrix, we need to add the batch prompting embeddings to the latent representations to obtain $\varvec{\textrm{Z}}^{(b)} = \left[\varvec{z}^{(b)}_1,\varvec{z}^{(b)}_2, \cdots , \varvec{z}^{(b)}_m\right]^{\textrm{T}}\in \mathbb {R}^{m\times d}$:

21 $$\begin{aligned} \varvec{z}^{(b)}_i = \varvec{z}_i + \varvec{b}_{l_i}^{(2)}, i=1,2,\cdots , m. \end{aligned}$$

$\varvec{\textrm{Z}}^{(b)}$ is then input into the decoder to reconstruct gene expression matrix $\widehat{\textbf{X}}$.

### The complete spCLUE model

Based on the individual modules introduced above, the entire forward propagation process of spCLUE for multi-slice data is summarized below:

22 $$\begin{aligned} \left\{ \begin{array}{l} \varvec{\textrm{X}}^{(b)} = \varvec{\textrm{X}} - \textbf{E}^{(b)} \textbf{B}_1, \\ \widetilde{\textbf{X}} = \mathcal {A}\left( \varvec{\textrm{X}}^{(b)}\right) , \\ \varvec{\textrm{Z}}_* = \sigma \left( \mathcal {C}\left( \widetilde{\varvec{\textrm{A}}}_{*}\right) \left( \sigma \left( \mathcal {C}\left( \widetilde{\varvec{\textrm{A}}}_{*}\right) \widetilde{\textbf{X}} \textbf{W}_{11} \right) \right) \textbf{W}_{12}\right) , \\ \varvec{\textrm{H}}_{*} = \textrm{ReLU}\left( \varvec{\textrm{Z}}_{*}\textbf{W}_{21}\right) \textbf{W}_{22}, \\ \varvec{\textrm{Q}}_{*} = \textrm{softmax}\left( \textrm{ReLU}\left( \varvec{\textrm{Z}}_{*}\textbf{W}_{31}\right) \textbf{W}_{32}\right) , \\ \varvec{\alpha }_{*} = \textrm{Attention}_{*}\left( \varvec{\textrm{Z}}_{spa}, \varvec{\textrm{Z}}_{expr}\right) , \\ \varvec{\textrm{Z}} = \varvec{\alpha }_{spa}\odot \varvec{\textrm{Z}}_{spa} + \varvec{\alpha }_{expr}\odot \varvec{\textrm{Z}}_{expr}, \\ \varvec{\textrm{Z}}^{(b)} = \varvec{\textrm{Z}} + \textbf{E}^{(b)} \textbf{B}_2, \\ \widehat{\textbf{X}} = \textrm{ReLU}\left( \varvec{\textrm{Z}}^{(b)}\textbf{W}_{12}^{\textrm{T}}\right) \textbf{W}_{11}^{\textrm{T}}, \end{array} \right. \end{aligned}$$

where $\mathcal {A}(\cdot )$ denotes the data augmentation step defined in Eq. (8), and $\textbf{E}^{(b)} = [e^{(b)}_{il}]_{m\times L}$ denotes an indicator matrix of batch information:

23 $$\begin{aligned} e_{il}^{(b)} = \left\{ \begin{array}{l} 1, \text {if spot}\ i\ \text {belongs to batch}\ l; \\ 0, \text {otherwise}. \end{array}\right. \end{aligned}$$

Additionally, $\widetilde{\varvec{\textrm{A}}}_{*}, \textbf{Z}_*, \varvec{\textrm{H}}_*,\varvec{\textrm{Q}}_*,\ \text {and}\ \varvec{\alpha }_*$ denote the intermediate results under each view (spatial locations or expression levels). Notably, for data with a single slice, the batch embeddings are removed by setting $\textbf{B}_1 = \textbf{B}_2 = \textbf{0}$.

spCLUE is then trained with the loss function defined in Eq. (19) and optimized using the Adam [56] algorithm. The detailed hyperparameter settings used in this manuscript are provided in the Additional file 1: Supplementary Methods. Given the trained model, spot representations ($\varvec{\textrm{Z}}$) are finally extracted at the bottleneck layer, which are used to identify spatial domains. We apply the mclust algorithm [57] combined with a refinement strategy proposed in GraphST [27] to infer spatial clusters from the spot representations (see Additional file 1: Supplementary Methods).

## Supplementary information

Additional file 1. Supplementary materials which include supplementary methods, supplementary figures S1-S39, and supplementary tables S1-S4.

## Data Availability

The DLPFC dataset is available at http://spatial.libd.org/spatialLIBD/ [58]. The BRCA dataset and the MOB2 dataset can be downloaded from https://github.com/JinmiaoChenLab/SEDR_analyses/tree/master/data [59]. The MOB1 dataset is available at https://singlecell.broadinstitute.org/single_cell/study/SCP815/highly-sensitive-spatial-transcriptomics-at-near-cellular-resolution-with-slide-seqv2#study-summary [60]. The BARISTA dataset can be downloaded from http://sdmbench.drai.cn [61]. The MOSTA dataset is available at https://db.cngb.org/stomics/mosta/ [62]. The spCLUE method is implemented with Python and is freely available at its Github repository https://github.com/EnchantedJoy/spCLUE [63] and Zenodo https://doi.org/10.5281/zenodo.15504121 [64] under the MIT license.
