# Supplementary material for: spCLUE: a contrastive learning approach to unified spatial transcriptomics analysis across single-slice and multi-slice data
Source: Genome Biol. 2025 Jun 23;26:177. doi: 10.1186/s13059-025-03636-0 (PMC12183872; doi:10.1186/s13059-025-03636-0)
Supplement: Supplementary file 1 — Additional file 1. Supplementary materials which include supplementary methods, supplementary figures S1-S39, and supplementary tables S1-S4. [file 13059_2025_3636_MOESM1_ESM.pdf]

Supplementary Materials – "spCLUE: A contrastive learning approach to unified spatial transcriptomics analysis across single-slice and multi-slice data"

1 Supplementary Methods

1.1 Implementation details of spCLUE

The spCLUE software is available at *https://github.com/EnchantedJoy/spCLUE/*, and was applied as described in its tutorial. The number of principal components in data preprocessing was set to $n = 200$. The default number of neighbors selected to construct graphs was set to $K = 12$. In the construction of multi-view graph, the self-connection weight λ is default to $\lambda = 0.3$ as suggested in the CCST method [1]. We further assessed the impact of this parameter by comparing the ARI scores of spCLUE across a range of λ values (from 0 to 0.9 in increments of 0.1) on four datasets: DLPFC, BRCA, MOSTA, and BARISTA (Supplementary Figure S35). We observed that spCLUE achieved better clustering performance with $\lambda = 0.3$ in most cases, demonstrating the reasonableness and generalizability of this default setting. For users who wish to tune the parameter on their own datasets, we recommend starting with smaller values, as larger values (e.g., 0.8 or 0.9) tend to degrade performance. In graph contrastive learning, the noise intensity was set to $\alpha = 0.01$, and the masking probability (also known as the dropout probability) of the input feature was set to $p = 0.5$. A small-scale analysis of edge corruption probabilities was conducted on the DLPFC slices, indicating that $p_m = 0.4$ was a reasonable choice (Supplementary Figure S36). This value was also further validated on the other three datasets. In contrastive learning, τ_{ins} and τ_{cls} were both set to 0.2. We compared different values of the temperature parameter ranging between 0.1 and 1.0 on the DLPFC dataset, and found that setting the values to 0.2 yielded better results. Then, we further evaluated the impact of these settings on other datasets, which confirmed that 0.2 is a reasonable default setting (Supplementary Figure S37). For the loss function, the weight of the instance contrastive loss was also compared in a small-scale analysis of DLPFC slices, indicating $\kappa = 0.1$ as a reasonable choice. We then extended the analysis to the other three datasets, confirming the generalizability of this default setting (Supplementary Figure S38). The dimensions of the GCN layers were set to $d_1 = 64$ and $d = 24$ as the default. The dimensions of the instance projection heads were set as $d_2 =$

$f = d_3 = 24$. The dimension (N_c) of the output layer in the clustering projection head was set to the number of tissue layers or cell types when the ground truth is known; otherwise, it is fixed at 12. A sensitivity analysis was conducted to evaluate spCLUE's accuracy under different fixed values of N_c. While the median ARI score showed a slight expected decrease in the absence of true cluster numbers, spCLUE demonstrated robust performance across varying parameter settings (Supplementary Figure S39). The learning rate of spCLUE was set to 0.001 and the weight decay was set to 0.001.

Clustering. After obtaining spot embeddings from the trained spCLUE network, the mclust method [2] was applied to the embeddings extracted by spCLUE to obtain clustering results. In addition, as suggested in GraphST [3], a cluster refinement strategy was applied to data sequenced by 10x Visium and BaristaSeq technologies. To achieve a fair comparison of clustering performance, the same clustering and refinement approach was used for alternative spatial domain identification methods that do not have built-in clustering steps (see "Implementation of alternative methods" in Supplementary Methods). For applications presented in this work, the number of clusters was set to the annotated layer or cell type number (when the true number was known) or 12 (when the true number was unknown).

1.2 Evaluation metrics

We adopted adjusted Rand index (ARI)[4], normalized mutual information (NMI)[5], silhouette coefficient (SC)[6] and Calinski–Harabasz index (CH)[7] to assess clustering performance. For datasets with annotated spatial regions, ARI and NMI were used to quantify the consistency between the predicted labels and ground-truth labels. For datasets without given annotations, SC and CH were used to reflect the clustering structure of spot representations.

ARI calculates the similarity between assignments U and V on n spots (i.e. $|U| = |V| = n$), which is defined as:

$$\mathrm{ARI} = \frac{\binom{n}{2}(a+d) - [(a+b)(a+c) + (c+d)(b+d)]}{\binom{n}{2}^2 - [(a+b)(a+c) + (c+d)(b+d)]},$$

where a is the number of spot pairs assigned to the same category in U and V, b is the number of spot pairs assigned to the same category in U but differs in V, c is the number of spot pairs assigned to the same category in V but differs in U, d is the number of spot pairs assigned to different categories in V and U. ARI ranges in $[-1, 1]$ and a higher score implies a better clustering result.

NMI also measures the similarity between U and V, and is defined as:

$$\mathrm{NMI} = \frac{-2\sum_{p=1}^{c_U}\sum_{q=1}^{c_V} |U_p \bigcap V_q| \log \frac{n|U_p \bigcap V_q|}{|U_p|\cdot|V_q|}}{\sum_{p=1}^{c_U} |U_p| \log \frac{|U_p|}{n} + \sum_{q=1}^{c_V} |V_q| \log \frac{|V_q|}{n}},$$

where U_p consists of spots assigned to category p in U, V_q consists of spots assigned to category q in V, $|U_p|$ denotes the cardinality of U_p, and n is total number of spots.

c_U and c_V denote the number of clusters in U and V, respectively. NMI value ranges in $[0, 1]$, and a higher score reflects better performance.

SC is a metric used to evaluate the quality of clustering results. It measures how similar a data point is to its own cluster compared to other clusters. Based on spot representations and predicted labels, SC is defined as:

$$\mathrm{SC} = \frac{1}{n}\sum_{I=1}^{K}\sum_{\boldsymbol{x}_i\in\mathcal{C}_I}\frac{\min_{J\neq I}\frac{1}{|\mathcal{C}_J|}\sum_{\boldsymbol{x}_j\in\mathcal{C}_J}d(\boldsymbol{x}_i,\boldsymbol{x}_j)-\frac{1}{|\mathcal{C}_I|-1}\sum_{\boldsymbol{x}_j\in\mathcal{C}_I}d(\boldsymbol{x}_i,\boldsymbol{x}_j)}{\max\left(\frac{1}{|\mathcal{C}_I|-1}\sum_{\boldsymbol{x}_j\in\mathcal{C}_I}d(\boldsymbol{x}_i,\boldsymbol{x}_j),\min_{J\neq I}\frac{1}{|\mathcal{C}_J|}\sum_{\boldsymbol{x}_j\in\mathcal{C}_J}d(\boldsymbol{x}_i,\boldsymbol{x}_j)\right)},$$

where $\mathcal{C}_I$ represents cluster I, K is the number of clusters, and $d(\cdot,\cdot)$ denotes the Euclidean distance function. SC ranges in $[-1, 1]$, and a value closer to 1 indicates more reasonable clusters.

The CH index evaluates clustering results by comparing tightness within a cluster and separation between clusters. It is calculated as:

$$\mathrm{CH} = \frac{n-K}{K-1}\cdot\frac{\sum_{I=1}^{K}\left|\mathcal{C}_I\right|\cdot d(\boldsymbol{c}_I,\boldsymbol{c})}{\sum_{I=1}^{K}\sum_{\boldsymbol{x}_i\in\mathcal{C}_I}d(\boldsymbol{x}_i,\boldsymbol{c}_I)},$$

where $\boldsymbol{c}_I = \frac{1}{|\mathcal{C}_I|}\sum_{\boldsymbol{x}_i\in\mathcal{C}_I}\boldsymbol{x}_i$ is the centroid of cluster $\mathcal{C}_I$, $\boldsymbol{c}$ is the overall centroid calculated as $\boldsymbol{c} = \frac{1}{n}\sum_{i=1}^{n}\boldsymbol{x}_i$ and n is the number of spots. CH ranges in $[0, +\infty]$, and a larger CH score reflects more distinguishable clustering results.

1.3 Implementation of alternative methods

To benchmark the performance of spCLUE, the following alternative methods were compared in the real data applications. For spatial domain identification, the Seurat [8], BayesSpace [9], BASS [10], SpaGCN [11], and Harmony [12] methods have built-in clustering methods, so their outputted cluster labels were directly used. For the other methods, the mclust method [2] was used to obtain the clustering labels based on the learned spot embeddings. Additionally, the same refinement strategy was applied to data sequenced by 10x Visium and BaristaSeq technologies.

Seurat [8] is a clustering method that only uses gene expression data. Seurat was applied as described in its online tutorial (*https://satijalab.org/seurat/articles/pbmc3k_tutorial.html*).

BayesSpace[9] is a Bayesian statistical model for clustering spatial transcriptomic data. It was applied as described in its online tutorial (*http://www.ezstatconsulting.com/BayesSpace/articles/BayesSpace*).

BASS [10] is a Bayesian statistical model that can handle both single-slice and multi-slice data. It was applied as described in its online tutorial (*https://zhengli09.github.io/BASS-Analysis*).

SpaceFlow [13] is a deep-learning-based method with a deep graph infomax (DGI) structure. It was applied as described in its online tutorial (*https://github.com/hongleir/SpaceFlow*).

SpaGCN [11] is a graph convolutional network model with deep embedded clustering strategy. It was applied as described in its online tutorial (*https://github.com/jianhuupenn/SpaGCN*).

CCST [1] is a deep-learning-based method with DGI structure. It was applied as described in its online tutorial (*https://github.com/xiaoyeye/CCST*).

STAGATE[14] is a deep-learning-based method with a graph attention network (GAT)[15] structure. It was applied as described in its online tutorial (*https://stagate.readthedocs.io/en/latest*).

GraphST [3] is a DGI-based method with a GCN structure. It was applied as described in its online tutorial (*https://deepst-tutorials.readthedocs.io/en/latest/*).

SEDR [16] is a deep-learning-based method with a variational graph auto-encoder (VGAE) structure. It was applied as described in its online tutorial (*https://sedr.readthedocs.io/en/latest*).

Harmony [12] is a non-spatial integration method. It was applied as described in its online tutorial (*https://github.com/slowkow/harmonypy*).

STAligner [17] is a GAT model with contrastive learning. It was applied as described in its online tutorial (*https://staligner.readthedocs.io/en/latest*).

1.4 Datasets

Datasets used in this study are all publicly available and were sequenced by different platforms, including 10x Visium, BaristaSeq, SlideSeq-V2 and Stereo-seq. A brief summary of these datasets are provided in Supplementary Table S2.

The DLPFC dataset was sequenced by the 10x Visium technology. The data is accessible from the spatialLIBD package (*http://spatial.libd.org/spatialLIBD/*). This dataset consists of 12 slices from 3 adult samples with 4 adjacent slices from each sample:

- Sample1: 151507,151508,151509, 151510;
- Sample2: 151669,151670,151671, 151672;
- Sample3: 151673,151674,151675, 151676.

Five spatial domains are annotated in 151669, 151670, 151671 and 151672, and seven spatial domains are annotated in the other 8 slices.

The BRCA dataset was sequenced by the 10x Visium technology. The data is accessible at *https://www.10xgenomics.com/* and was annotated into 20 spatial domains in the SEDR work. The data and the corresponding labels are available at *https://github.com/JinmiaoChenLab/SEDR_analyses/tree/master/data*.

The MOSTA dataset was sequenced by the Stereo-seq technology. It consists of five mouse embryo slices at the developmental stage of E9.5. The MOSTA dataset is available at *https://db.cngb.org/stomics/mosta/*.

The BARISTA dataset was sequenced by the BaristaSeq technology. It consists of three slices of mouse primary visual cortex samples. The data is available at *http://sdmbench.drai.cn*.

The MOB1 dataset was sequenced by the Slide-seqV2 technology. The data is available at the Broad Institute Single Cell Portal at *https://singlecell.broadinstitute.org/single_cell/study/SCP815/highly-sensitive-spatial-transcriptomics-at-near-cellular-resolution-with-slide-seqv2#study-summary*.

The MOB2 dataset was sequenced by Stereo-seq technology. The data is available at *https://github.com/JinmiaoChenLab/SEDR_analyses/tree/master/data*.

1.5 Differential gene expression analysis

For the MOB datasets, we performed differential gene expression analysis to better understand the identified spatial domains by spCLUE. Differentially expressed genes (DEGs) were identified using the Wilcoxon rank-sum test to compare gene expression levels between cells in each cluster and all other cells. Meanwhile, a threshold of 0.05 was applied to the false discovery rate to correct for multiple hypothesis testing. Finally, top three genes with smallest adjusted P-value were selected for each cluster to be compared with known marker genes. The DEG analysis was implemented with the *scanpy.tl.rank_genes_groups* function in the Scanpy package [18].

1.6 Simulation design

We simulated three datasets using the Splatter package [19], which uses the gamma-Poisson distribution to model the gene expression. Each simulated dataset used one multi-slice dataset as the reference and had the same spatial locations and ground-truth labels as the reference dataset (Supplementary Table S4). Then, we selected one slice from each reference dataset and used the Splatter package to learn domain-specific gene expression distributions. Next, we generated synthetic gene expression data for each slice in the simulated dataset, based on the learned expression distributions. Importantly, batch effects were introduced by applying multiplicative scaling factors to the mean gene expression levels. These factors were consistent across spots within the same slice but varied between slices, thereby creating controlled batch effects across the simulated multi-slice data. The simulation was implemented with the *splatSimulate* function in the Splatter package with both location and scale parameters of the log normal distribution set to 0.8.

1.7 Calculation of LS and LE

To explore the performance of spCLUE across different datasets, we utilizes label smoothness (LS) and label entropy (LE) to summarize each dataset.

LS describes how similar the annotated spatial domains are between neighboring spots in a dataset. If nearby spots tend to be assigned the same label, the smoothness is high. If neighboring spots often have different labels, the smoothness is low. The value of LS ranges from 0 to 1, with higher values indicating more consistent labeling across nearby spots. For a single slice, it is calculated as:

$$\mathrm{LS} = \frac{1}{mK} \sum_{i=1}^{m} \sum_{j \in \mathcal{N}_i} \mathbf{1}\{c_i = c_j\},$$

where m denotes number of spots, K (set to 12) is the number of neareast neighbors, $\mathcal{N}_i$ denotes the K nearest neighbors of spot i, and c_i denotes the domain label of spot i.

LE measures how evenly the labels are distributed across the tissue. If each label appears in roughly equal proportions, the entropy is high. If only a few labels dominate while others are rare, the entropy is low. This measure helps assess whether the annotated spatial domains are balanced or skewed toward a few dominant labels. The value of LE usually ranges from 0 to a dataset-specific maximum that depends on the number of unique labels, with higher values indicating more diversity in the label distribution. For a single slice, it is calculated as:

$$\text{LE} = -\frac{1}{m}\sum_{i=1}^{m}\sum_{d=1}^{N_d} p_{id}\log p_{id},$$

where $p_{id} = \sum_{j\in\mathcal{N}_i}\mathbf{1}\{c_j = d\}/K$ denotes the proportion of neighboring spots of spot i that belong to domain d, and N_d denotes number of annotated spatial domains.

References

[1] Jiachen Li, Siheng Chen, Xiaoyong Pan, Ye Yuan, and Hong-Bin Shen. Cell clustering for spatial transcriptomics data with graph neural networks. *Nature Computational Science*, 2(6):399–408, 2022.

[2] Chris Fraley and Adrian E Raftery. Enhanced model-based clustering, density estimation, and discriminant analysis software: Mclust. *Journal of Classification*, 20(2):263–286, 2003.

[3] Yahui Long, Kok Siong Ang, Mengwei Li, Kian Long Kelvin Chong, Raman Sethi, Chengwei Zhong, Hang Xu, Zhiwei Ong, Karishma Sachaphibulkij, Ao Chen, et al. Spatially informed clustering, integration, and deconvolution of spatial transcriptomics with GraphST. *Nature Communications*, 14:1155, 2023.

[4] Lawrence Hubert and Phipps Arabie. Comparing partitions. *Journal of Classification*, 2(1):193–218, 1985.

[5] Alexander Strehl and Joydeep Ghosh. Cluster ensembles—a knowledge reuse framework for combining multiple partitions. *Journal of Machine Learning Research*, 3(Dec):583–617, 2002.

[6] Peter J Rousseeuw. Silhouettes: a graphical aid to the interpretation and validation of cluster analysis. *Journal of Computational and Applied Mathematics*, 20:53–65, 1987.

[7] Tadeusz Caliński and Jerzy Harabasz. A dendrite method for cluster analysis. *Communications in Statistics*, 3(1):1–27, 1974.

[8] Tim Stuart, Andrew Butler, Paul Hoffman, Christoph Hafemeister, Efthymia Papalexi, William M Mauck III, Yuhan Hao, Marlon Stoeckius, Peter Smibert, and Rahul Satija. Comprehensive integration of single-cell data. *Cell*, 177(7):1888–1902, 2019.

[9] Edward Zhao, Matthew R Stone, Xing Ren, Jamie Guenthoer, Kimberly S Smythe, Thomas Pulliam, Stephen R Williams, Cedric R Uytingco, Sarah EB Taylor, Paul Nghiem, et al. Spatial transcriptomics at subspot resolution with BayesSpace. *Nature Biotechnology*, 39(11):1375–1384, 2021.

[10] Zheng Li and Xiang Zhou. Bass: multi-scale and multi-sample analysis enables accurate cell type clustering and spatial domain detection in spatial transcriptomic studies. *Genome biology*, 23(1):168, 2022.

[11] Jian Hu, Xiangjie Li, Kyle Coleman, Amelia Schroeder, Nan Ma, David J Irwin, Edward B Lee, Russell T Shinohara, and Mingyao Li. SpaGCN: Integrating gene expression, spatial location and histology to identify spatial domains and spatially variable genes by graph convolutional network. *Nature Methods*, 18(11):1342–1351, 2021.

[12] Ilya Korsunsky, Nghia Millard, Jean Fan, Kamil Slowikowski, Fan Zhang, Kevin Wei, Yuriy Baglaenko, Michael Brenner, Po-ru Loh, and Soumya Raychaudhuri. Fast, sensitive and accurate integration of single-cell data with harmony. *Nature Methods*, 16(12):1289–1296, 2019.

[13] Honglei Ren, Benjamin L Walker, Zixuan Cang, and Qing Nie. Identifying multicellular spatiotemporal organization of cells with SpaceFlow. *Nature Communications*, 13:4076, 2022.

[14] Kangning Dong and Shihua Zhang. Deciphering spatial domains from spatially resolved transcriptomics with an adaptive graph attention auto-encoder. *Nature Communications*, 13(1):1739, 2022.

[15] Petar Veličković, Guillem Cucurull, Arantxa Casanova, Adriana Romero, Pietro Lio, and Yoshua Bengio. Graph attention networks. *arXiv:1710.10903*, 2017.

[16] Hang Xu, Huazhu Fu, Yahui Long, Kok Siong Ang, Raman Sethi, Kelvin Chong, Mengwei Li, Rom Uddamvathanak, Hong Kai Lee, Jingjing Ling, et al. Unsupervised spatially embedded deep representation of spatial transcriptomics. *Genome Medicine*, 16(1):12, 2024.

[17] Xiang Zhou, Kangning Dong, and Shihua Zhang. Integrating spatial transcriptomics data across different conditions, technologies and developmental stages. *Nature Computational Science*, 3(10):894–906, 2023.

[18] F Alexander Wolf, Philipp Angerer, and Fabian J Theis. Scanpy: large-scale single-cell gene expression data analysis. *Genome Biology*, 19:15, 2018.

[19] Luke Zappia, Belinda Phipson, and Alicia Oshlack. Splatter: simulation of single-cell rna sequencing data. *Genome biology*, 18(1):174, 2017.

2 Supplementary Figures

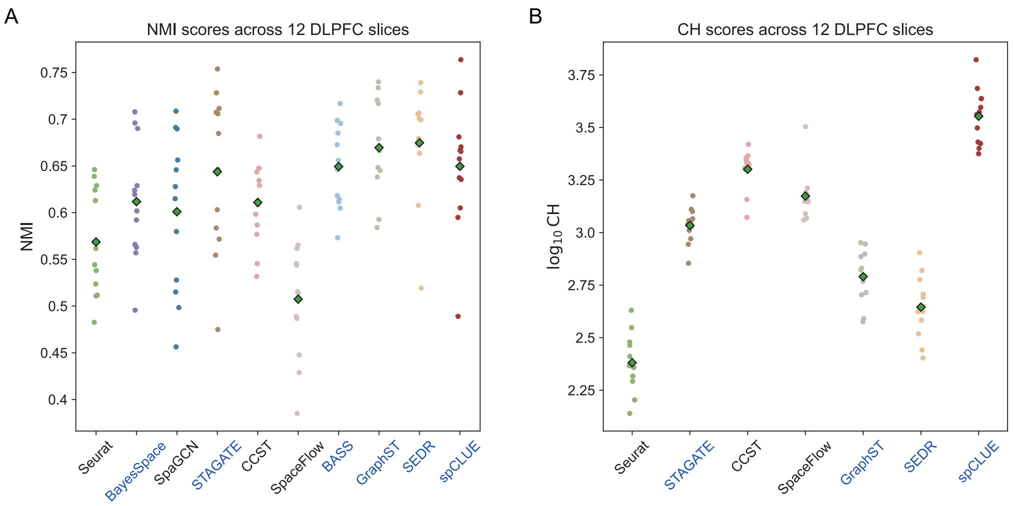

Fig S1 Comparison of spatial domain identification methods on 12 DLPFC slices. (A) NMI scores. (B) CH index. In each plot, the average values of scores are highlighted with the diamond marks.

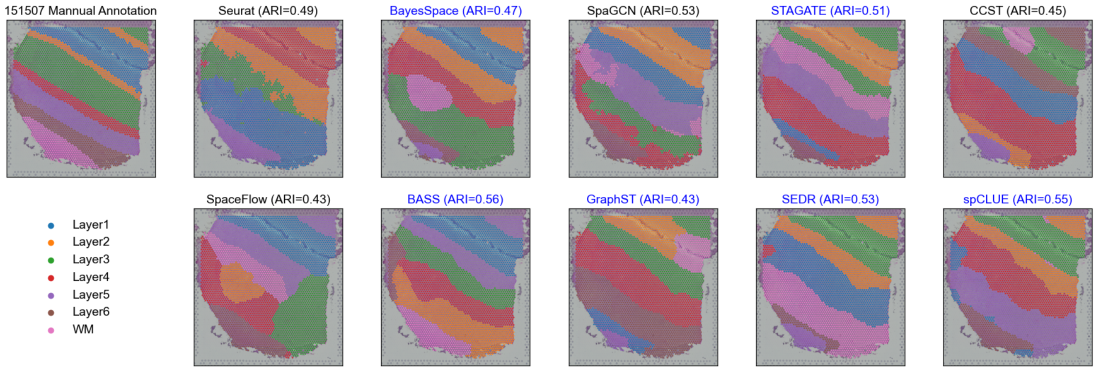

Fig S2 Visualization of clustering results on slice 151507 .

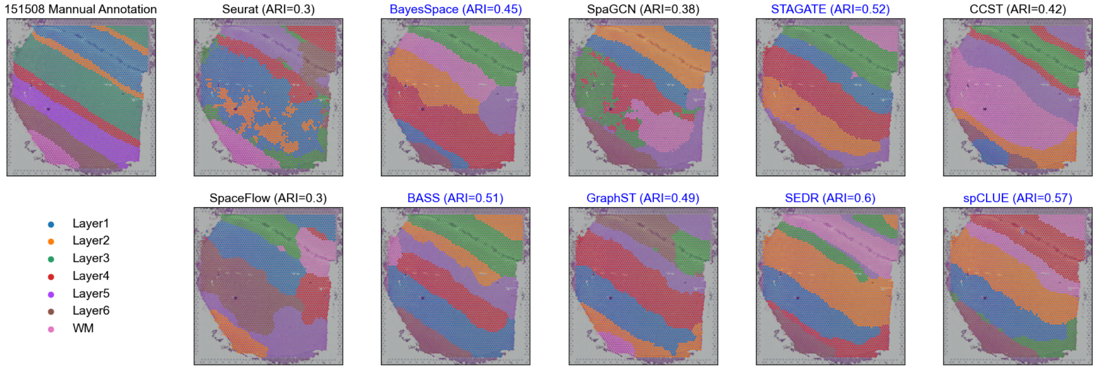

Fig S3 Visualization of clustering results on slice 151508.

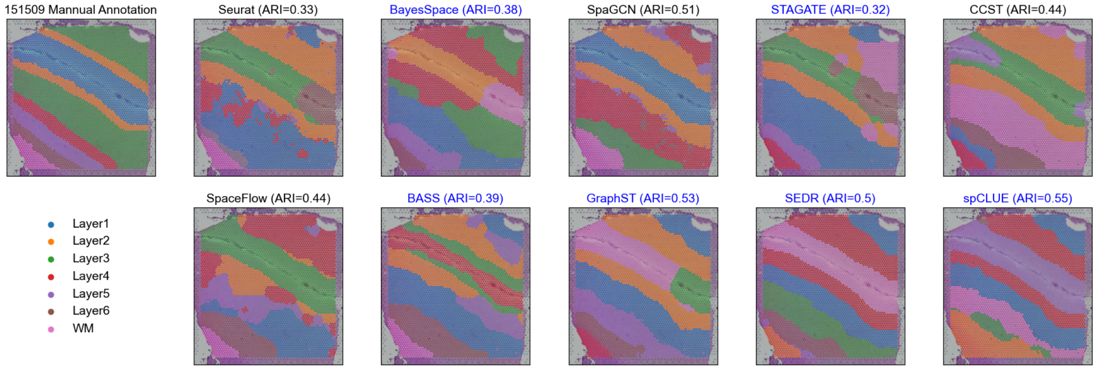

Fig S4 Visualization of clustering results on slice 151509.

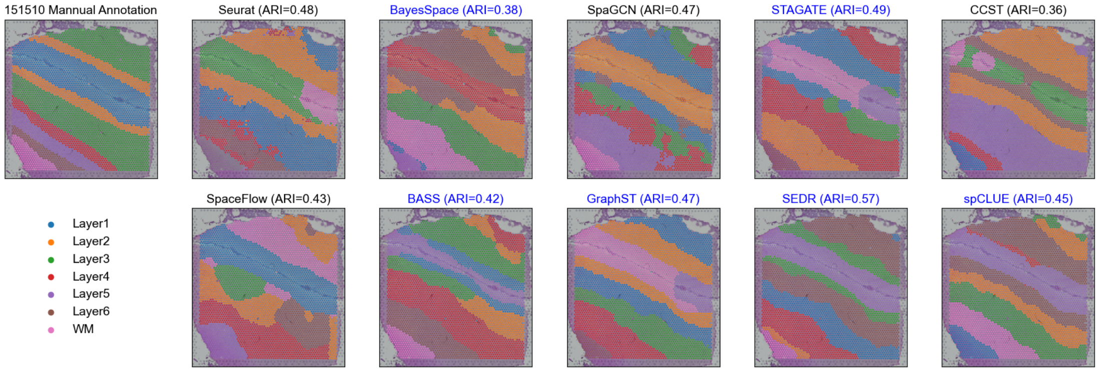

Fig S5 Visualization of clustering results on slice 151510.

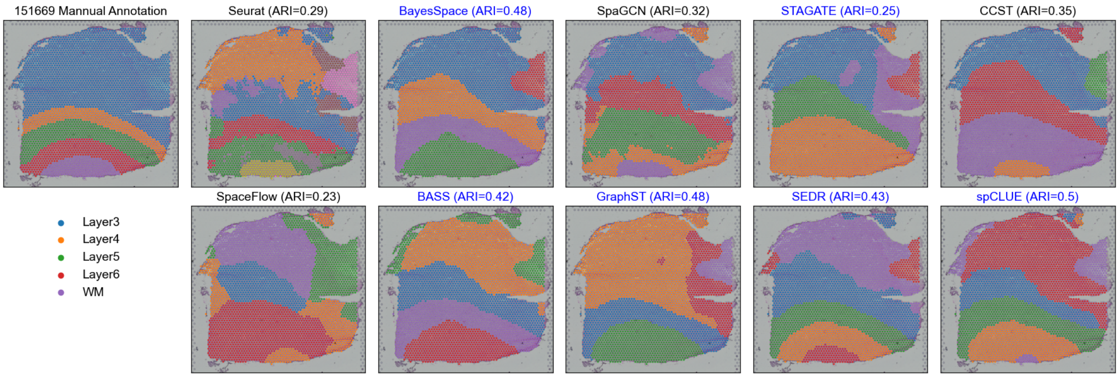

Fig S6 Visualization of clustering results on slice 151669.

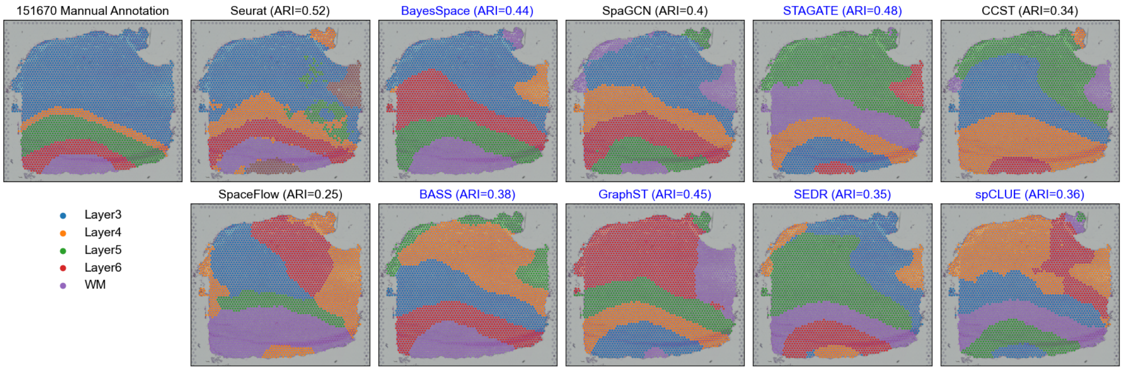

Fig S7 Visualization of clustering results on slice 151670.

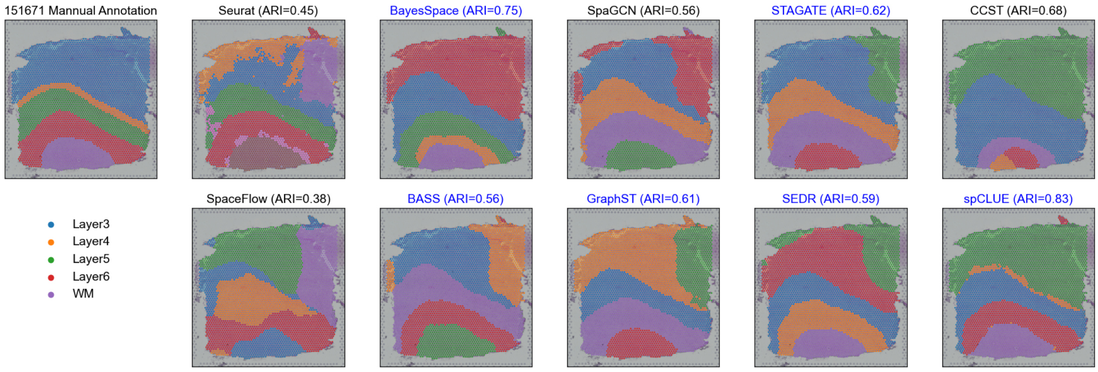

Fig S8 Visualization of clustering results on slice 151671.

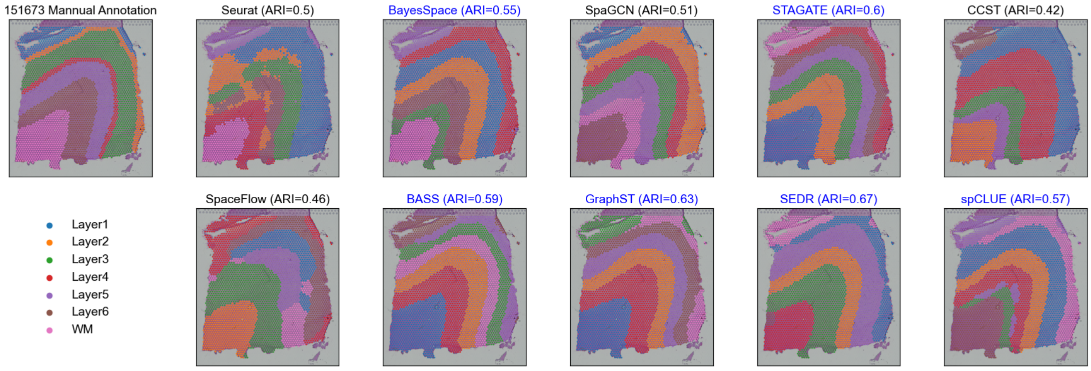

Fig S9 Visualization of clustering results on slice 151673.

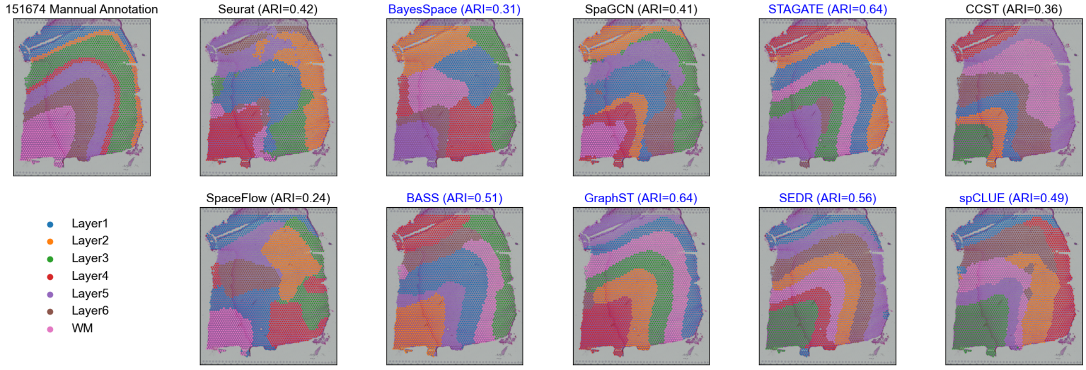

Fig S10 Visualization of clustering results on slice 151674.

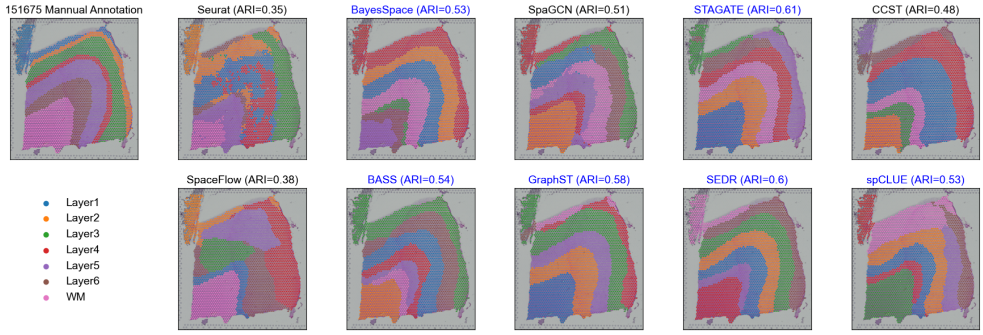

Fig S11 Visualization of clustering results of each method on slice 151675.

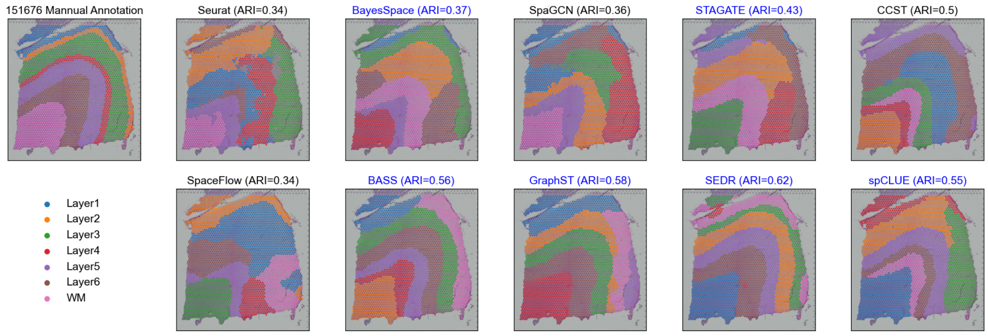

Fig S12 Visualization of clustering results of each method on slice 151676.

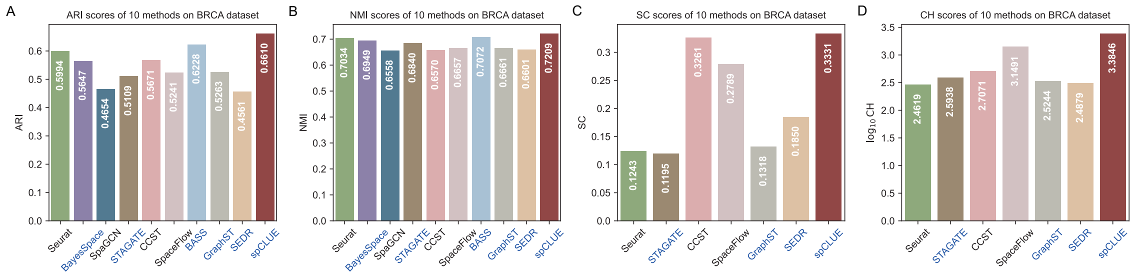

Fig S13 Comparison of ARI, NMI, SC, and CH scores of ten spatial domain identification methods on the BRCA dataset. (A) ARI scores. (B) NMI scores. (C) SC scores. (D) CH scores.

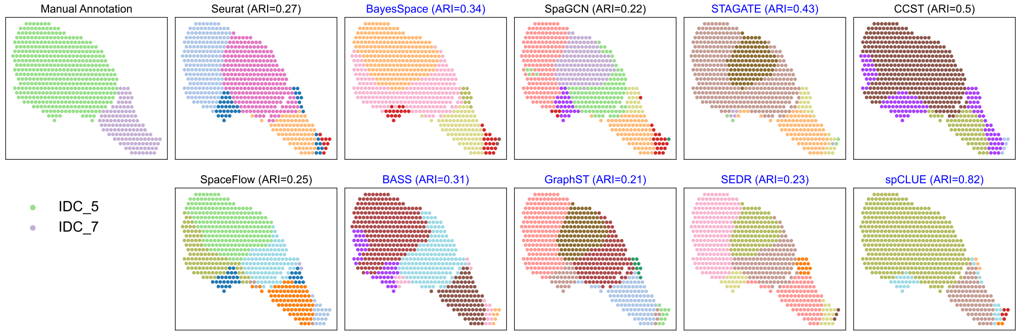

Fig S14 Comparison of inferred clusters by ten spatial domain identification methods on IDC_5 and IDC_7 regions of the BRCA dataset.

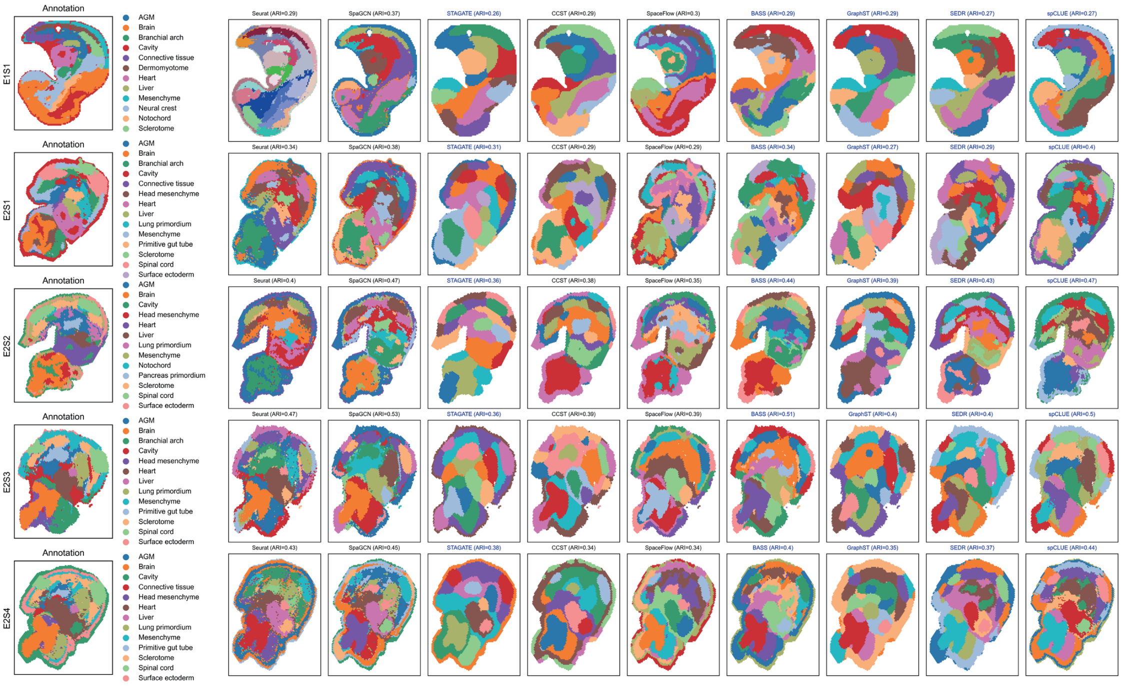

Fig S15 Comparison of spatial domain identification methods on the MOSTA dataset. Each row corresponds to one slice. The first column corresponds to the annotated labels and the rest columns correspond to different methods.

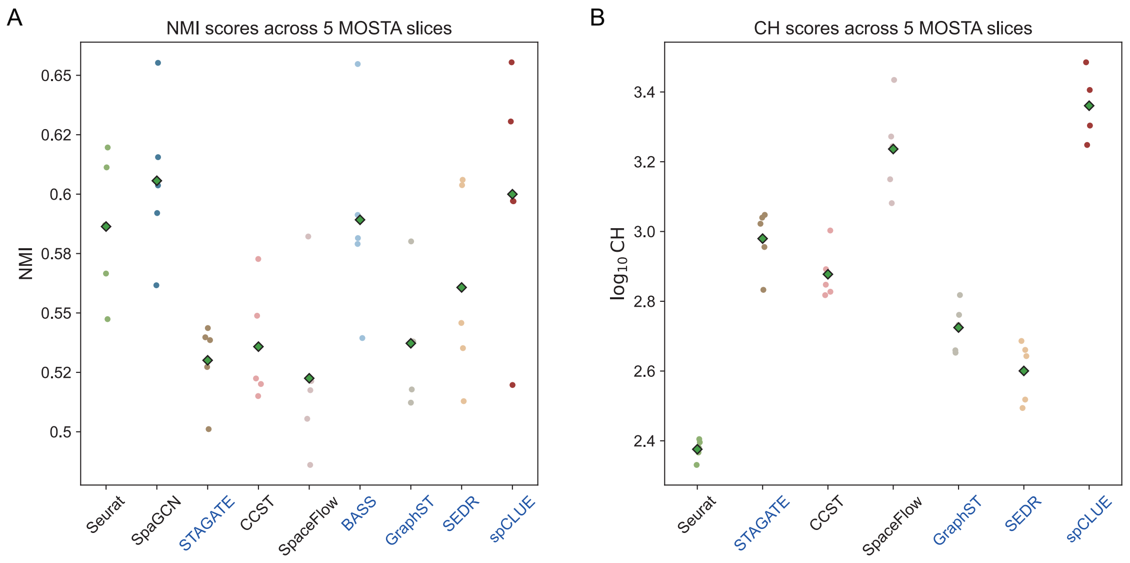

Fig S16 Comparison of NMI and CH scores on the five MOSTA slices. (A) NMI scores. (B) CH scores. CH scores were $\log_{10}$ transformed for presentation.

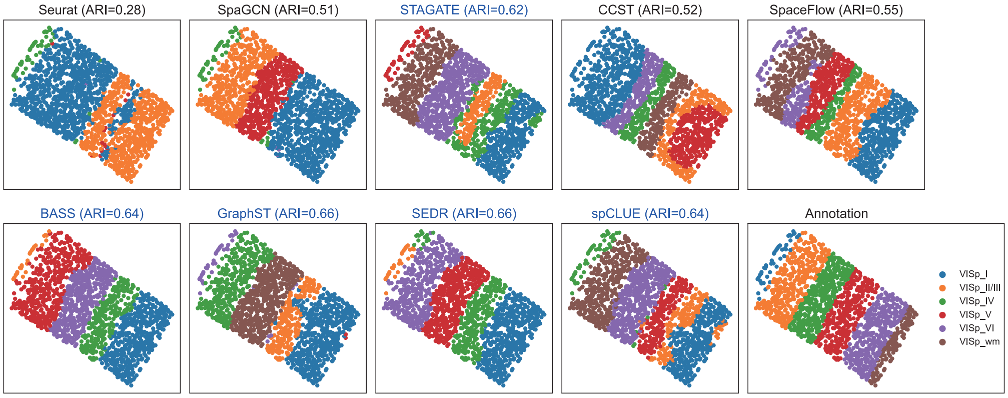

Fig S17 Visualization of spatial locations and inferred domains in slice 1 of the BARISTA dataset.

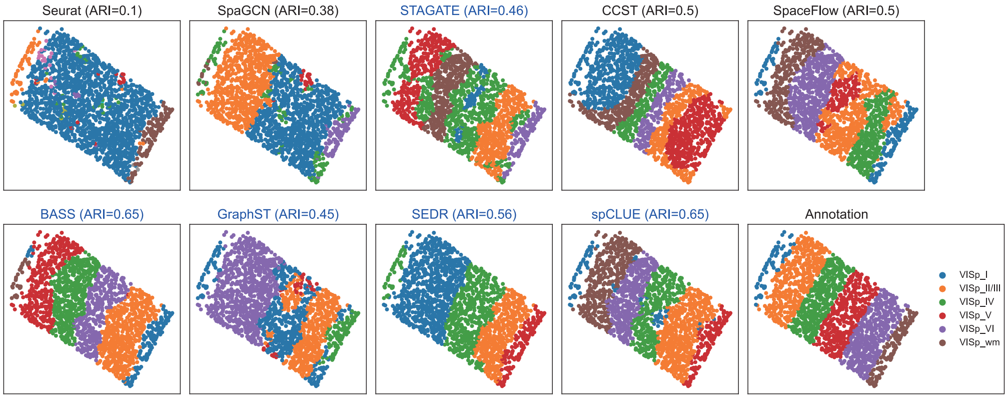

Fig S18 Visualization of spatial locations and inferred domains in slice 3 of the BARISTA dataset.

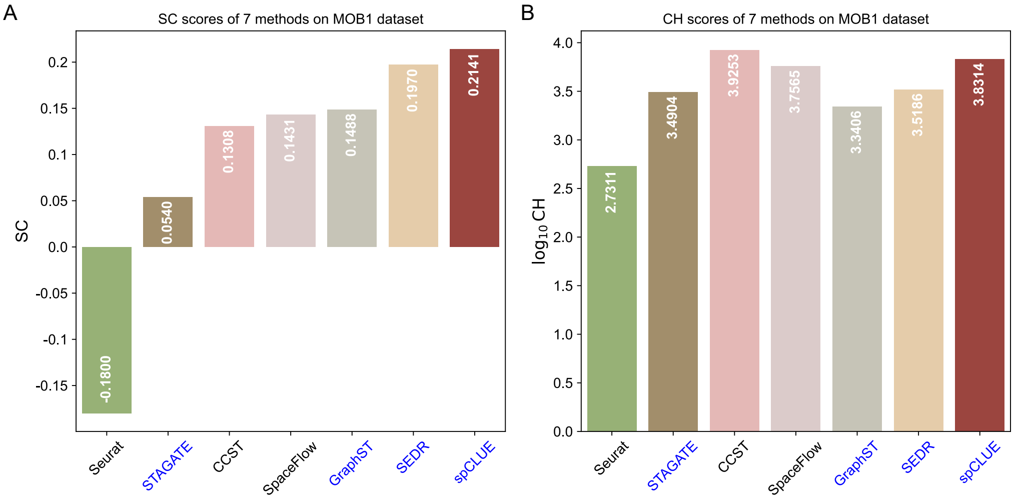

Fig S19 Comparison of SC (A) and CH (B) scores on the MOB1 dataset. CH scores were $\log_{10}$ transformed for presentation.

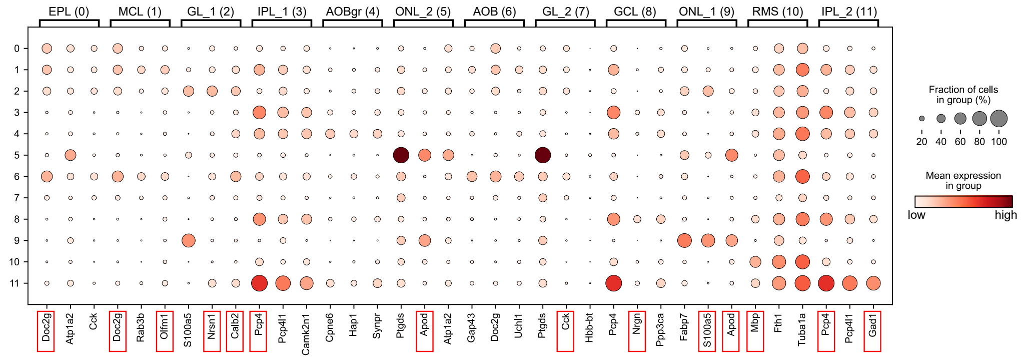

Fig S20 Top highly expressed genes in spatial domains inferred by spCLUE on the MOB1 dataset (Supplementary Methods). Known marker genes of specific domains are highlighted with red boxes.

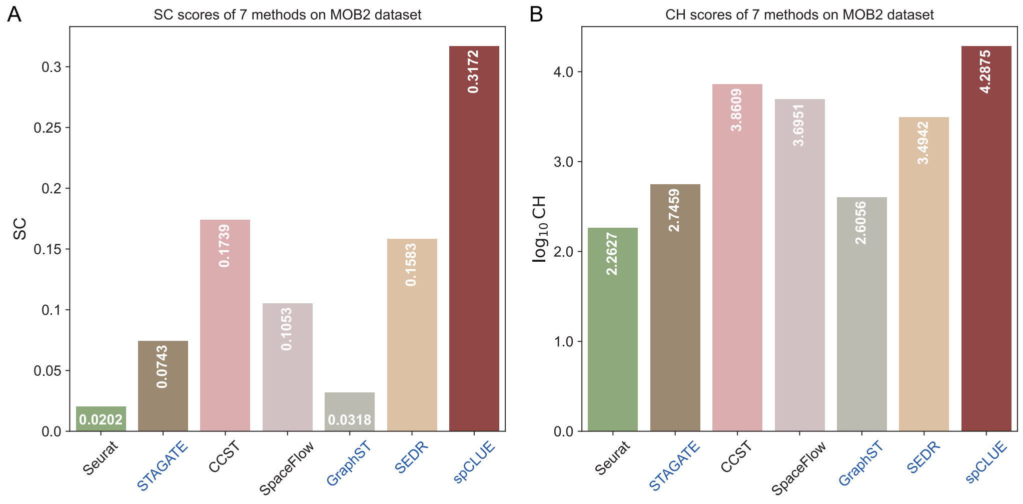

Fig S21 Comparison of SC and CH scores on the MOB2 dataset. (A) SC scores. (B) CH scores. CH scores were $\log_{10}$ transformed for presentation.

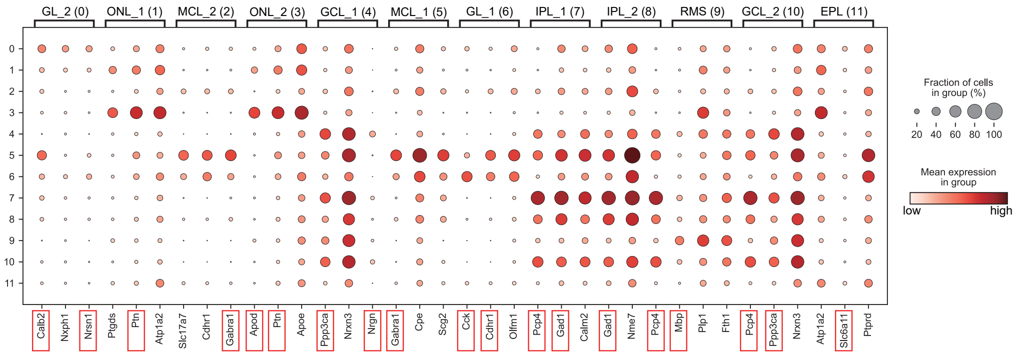

Fig S22 Top highly expressed genes in spatial domains inferred by spCLUE on the MOB2 dataset (Supplementary Methods). Known marker genes of specific domains are highlighted with red boxes.

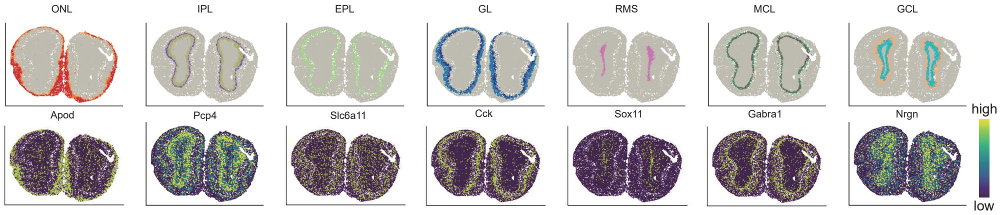

Fig S23 Known marker genes of spatial domains identified by spCLUE on the MOB2 dataset.

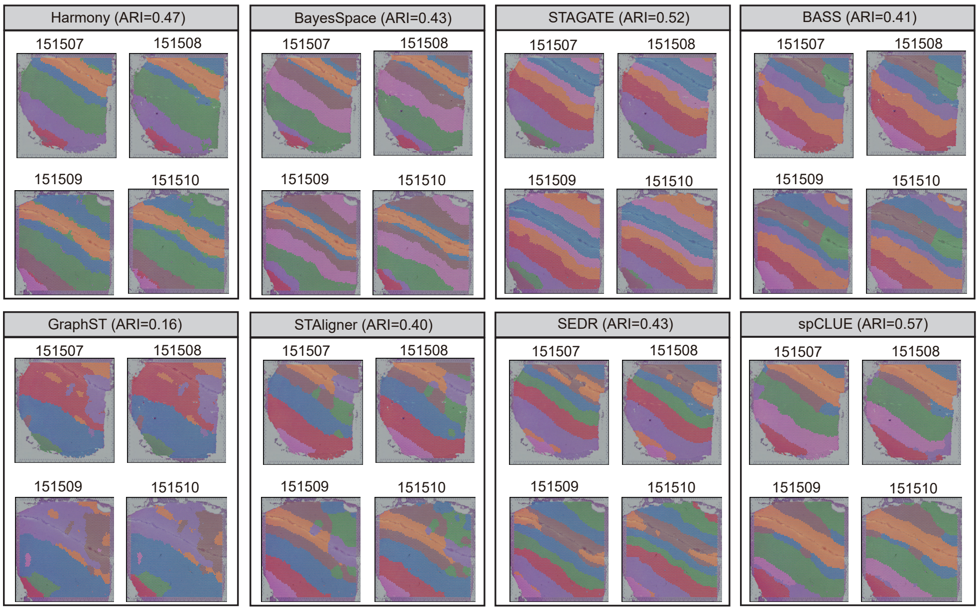

Fig S24 Comparison on DLPFC Sample 1.

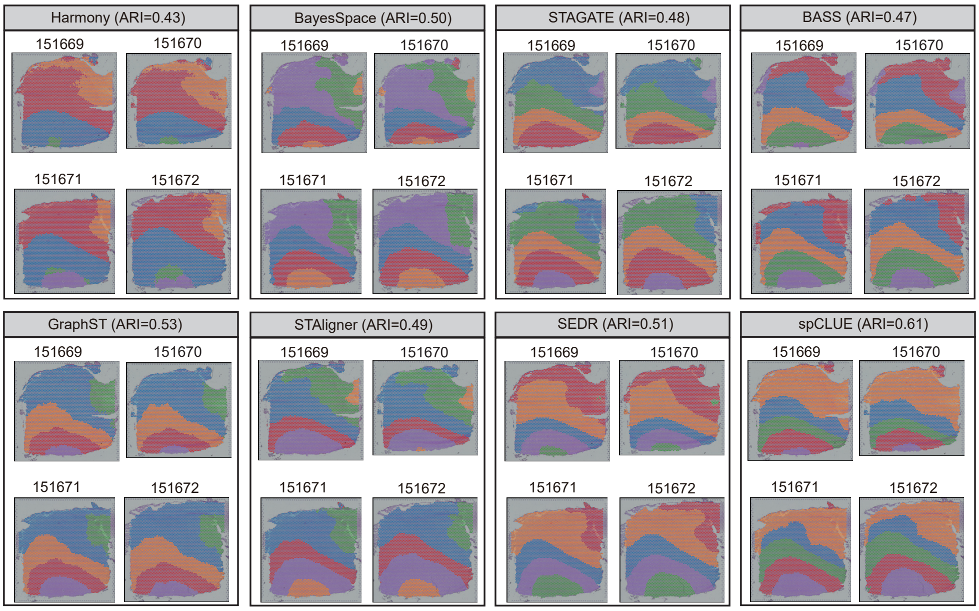

Fig S25 Comparison on DLPFC Sample 2.

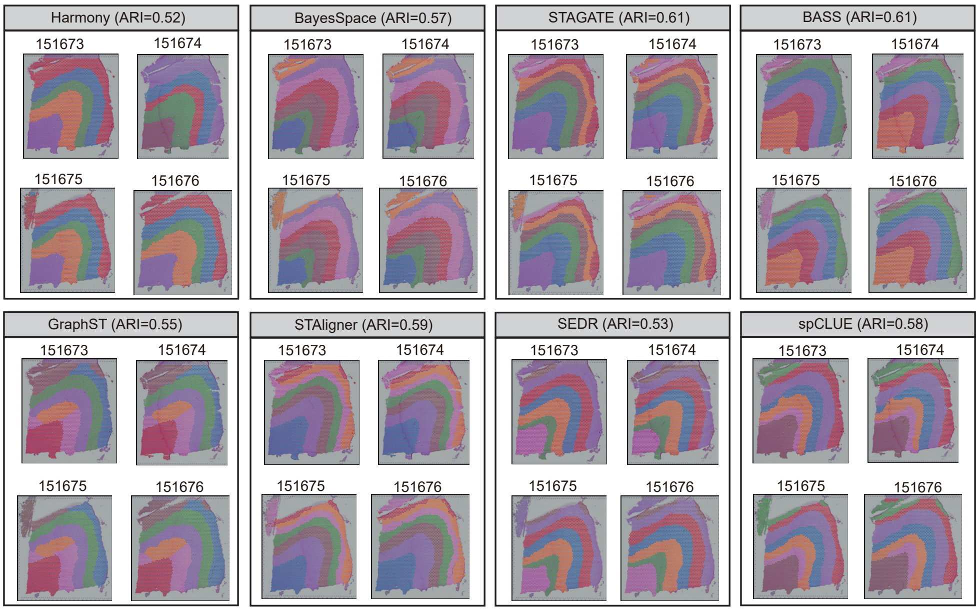

Fig S26 Comparison on DLPFC Sample 3.

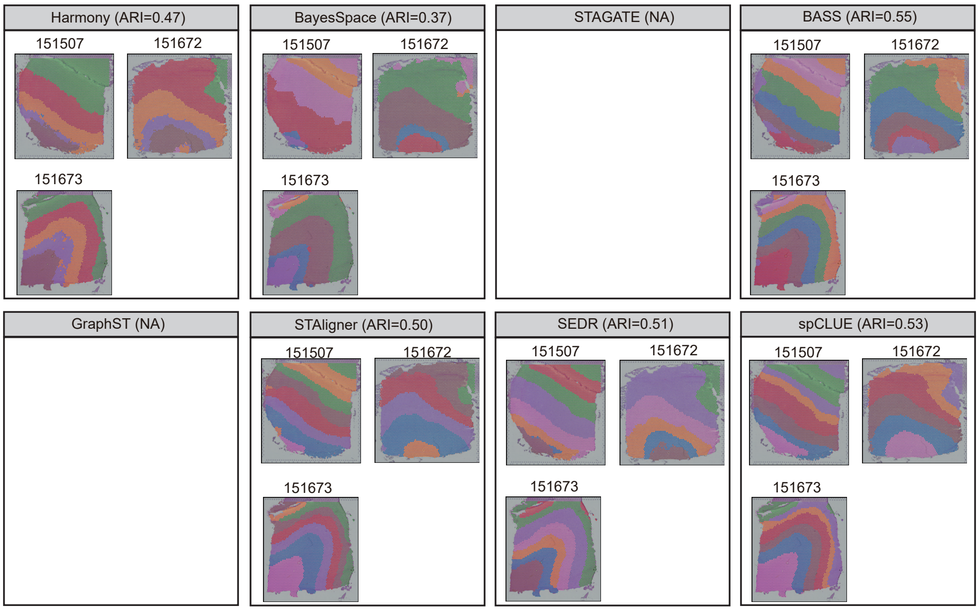

Fig S27 Comparison on DLPFC Sample 4.

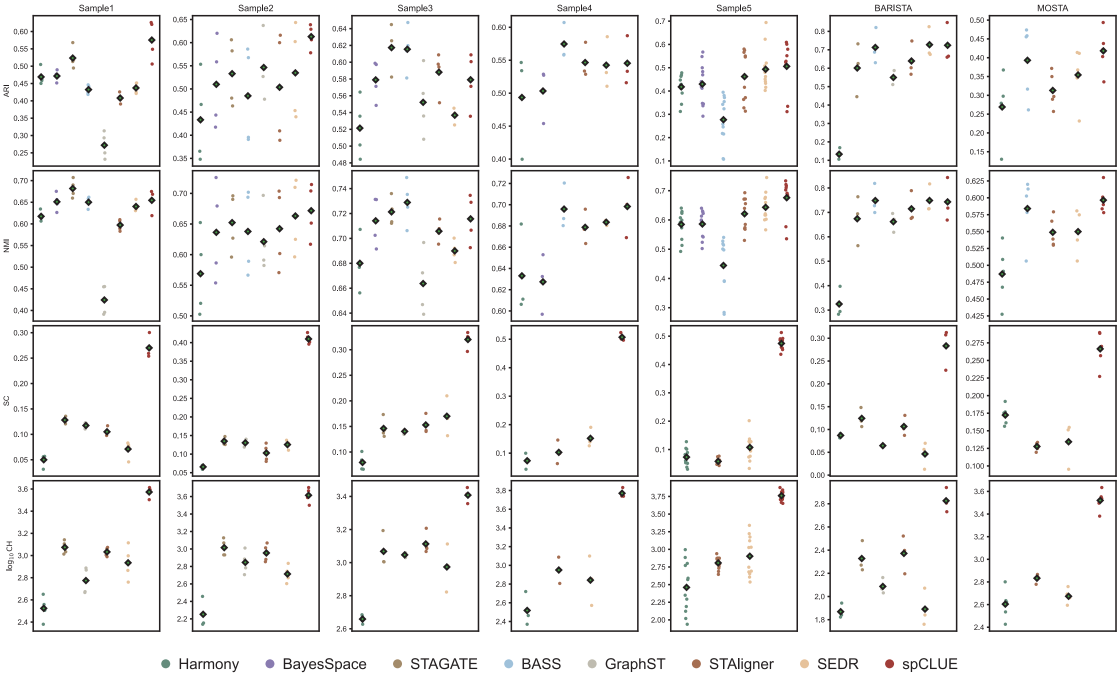

Fig S28 Comparison of ARI, NMI, SC, and CH scores on seven multi-slice datasets.

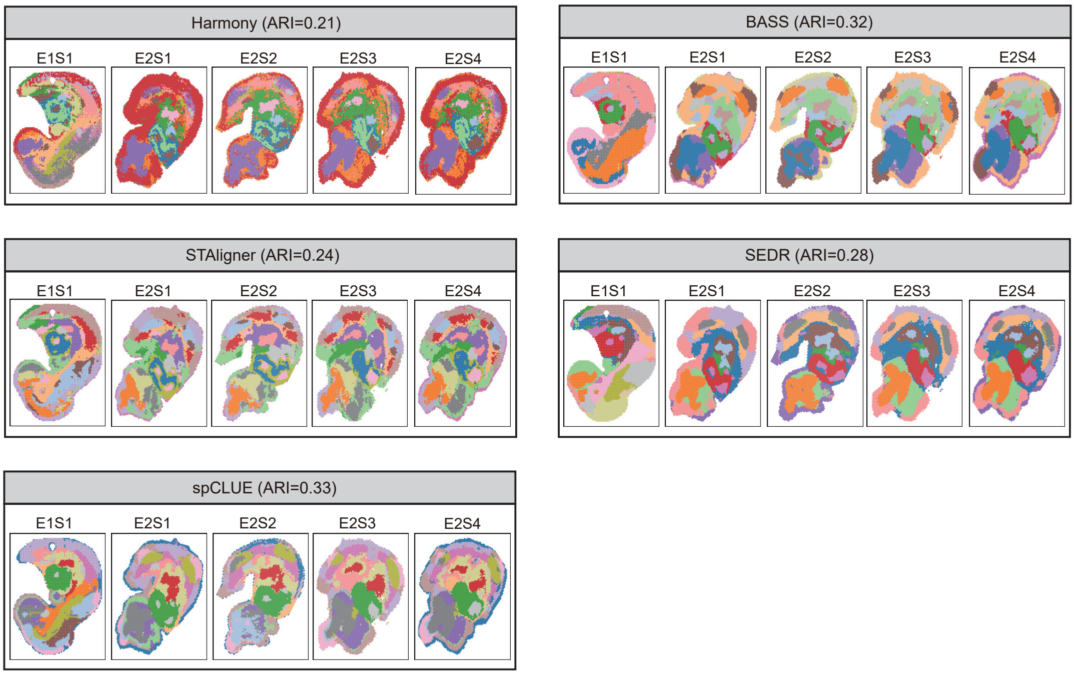

Fig S29 Multi-slice integration of the MOSTA dataset.

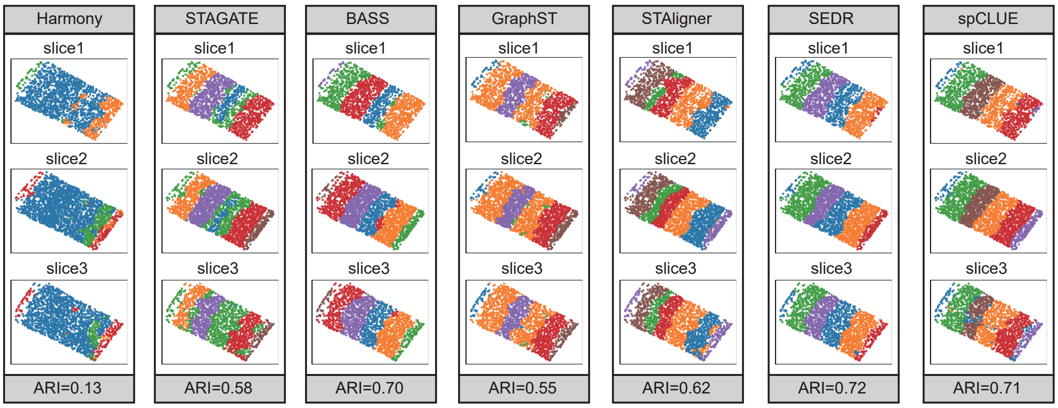

Fig S30 Multi-slice integration of the BARISTA dataset.

A

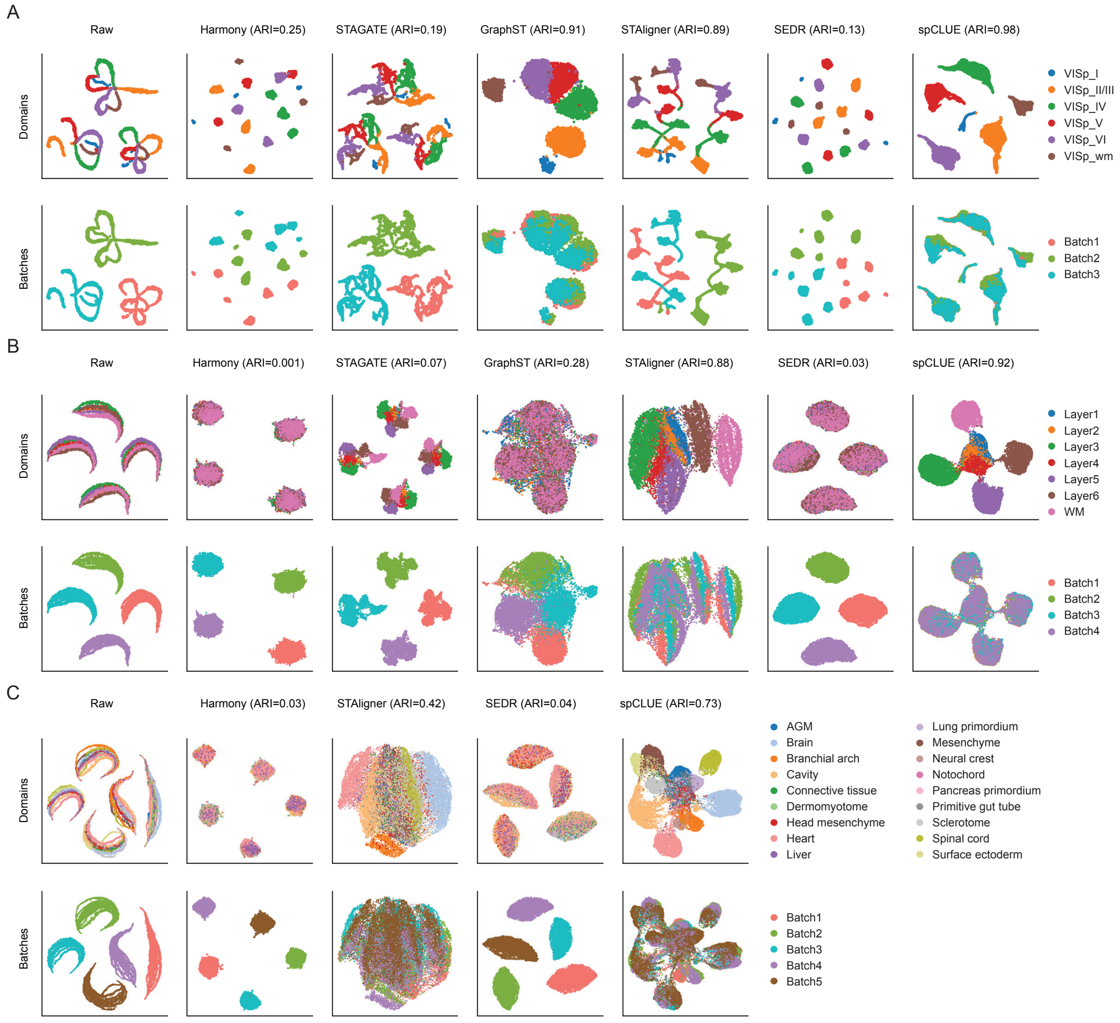

D

Method	BARISTAsim Batch correction	BARISTAsim Bio conservation	BARISTAsim Total	DLPFCsim Batch correction	DLPFCsim Bio conservation	DLPFCsim Total	MOSTAsim Batch correction	MOSTAsim Bio conservation	MOSTAsim Total	Ranking 1st	Ranking 2nd	Ranking 3rd
Harmony	0.15	0.52	0.37	0.09	0.31	0.22	0.08	0.37	0.25	0/3	0/3	1/3
STAGATE	0.11	0.48	0.33	0.08	0.42	0.28	NA	NA	NA	0/3	0/3	0/3
GraphST	0.45	0.42	0.43	0.25	0.31	0.29	NA	NA	NA	0/3	0/3	2/3
STAligner	0.22	0.79	0.56	0.42	0.57	0.51	0.37	0.52	0.46	0/3	3/3	0/3
SEDR	0.13	0.54	0.37	0.04	0.31	0.20	0.04	0.36	0.23	0/3	0/3	0/3
spCLUE	0.53	0.95	0.78	0.56	0.85	0.74	0.42	0.70	0.59	3/3	0/3	0/3

Fig S31 Comparison of multi-slice integration performance on simulated datasets. (A-C) UMAP visualization results of spot representations learned by different methods on the BARISTAsim (A), DLPFCsim (B), and MOSTAsim (C) dataset. UMAP plots labeled with "Raw" are obtained directly from the original gene expression data without any integration. In each panel, spots in the first row are colored by the spatial domains and those in nthe second row are colored by batch (i.e., slice) labels. STAGATE and GraphST were not applied on the MOSTAsim dataset since they require aligned slices. (D) Bio-conservation and batch-correction scores calculated using the scIB method. Both metrics range from 0 to 1, with 1 representing the best performance. The last three ranking columns show the frequency with which each method ranked among the top three positions across three datasets.

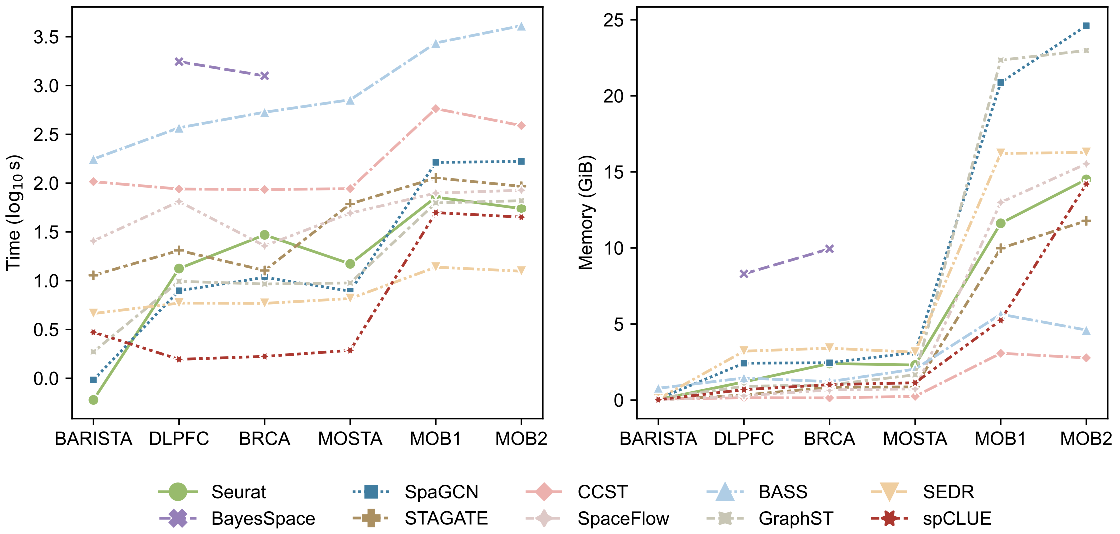

Fig S32 Comparison of running time (A) and memory cost (B) across single-slice datasets. Running time was $\log_{10}$ transformed for presentation. For datasets that include multiple slices, the average time or memory usage across slices is presented. BayesSpace was only evaluated on the DLPFC and BRCA datasets as it is specially designed for 10x and ST data.

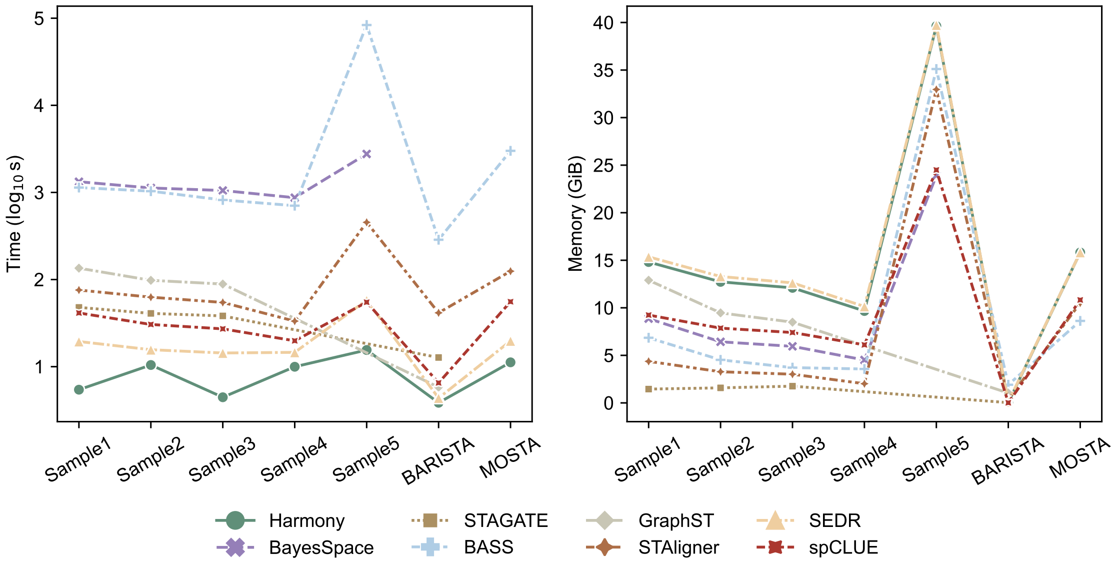

Fig S33 Comparison of running time (A) and memory cost (B) across multi-slice datasets. Running time was $\log_{10}$ transformed for presentation. BayesSpace was only evaluated on the DLPFC datasets as it is specially designed for 10x and ST data; STAGATE, and GraphST were only evaluated on Sample1, Sample2, Sample3, and BARISTA datasets as they are not applicable to unaligned slices.

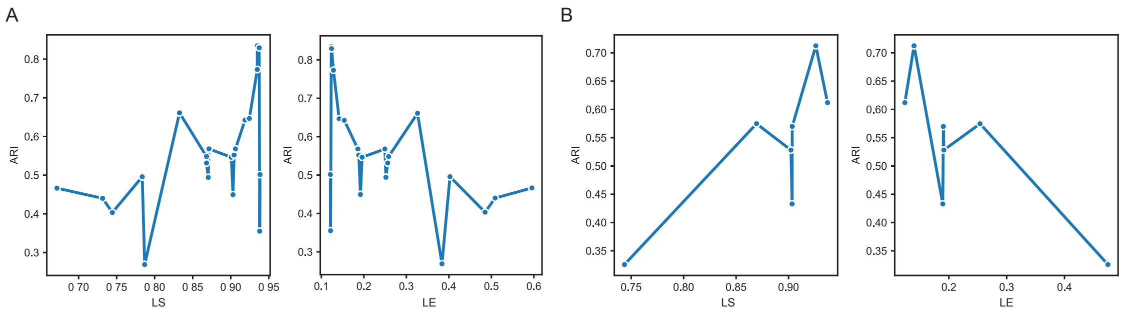

Fig S34 Comparison of spCLUE's performance in terms of LS and LE on single-slice (A) and multi-slice (B) datasets.

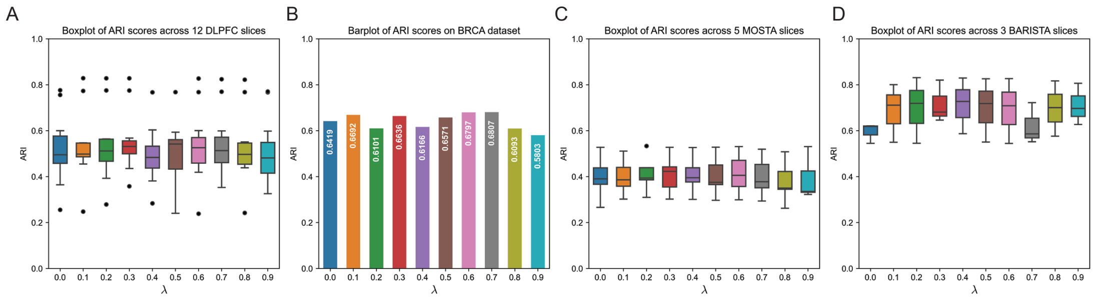

Fig S35 Performance of spCLUE with different values of λ on DLPFC (A), BRCA (B), MOSTA (C), and BARISTA (D) datasets.

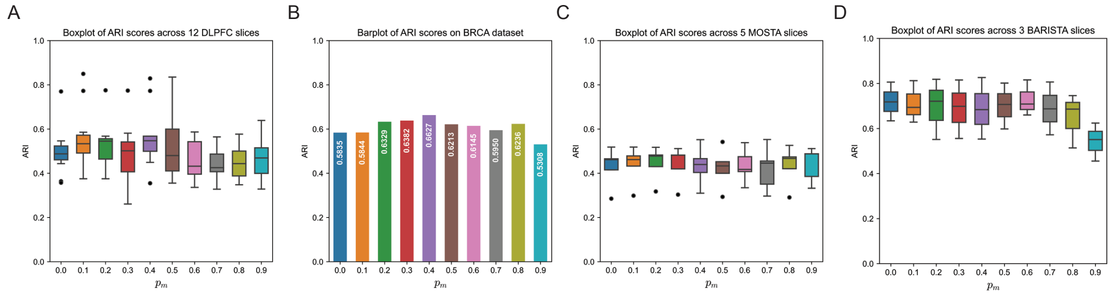

Fig S36 Performance of spCLUE with different values of p_m on DLPFC (A), BRCA (B), MOSTA (C), and BARISTA (D) datasets.

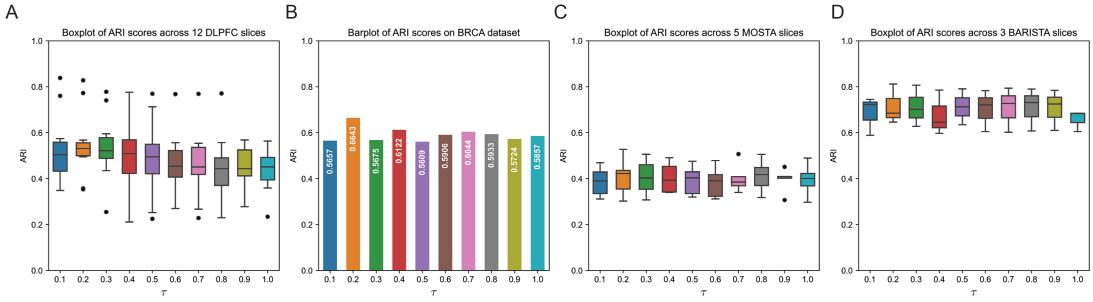

Fig S37 Performance of spCLUE across single-slice datasets under different settings of $\tau = \tau_{ins} = \tau_{cls}$. (A) DLPFC dataset. (B) BRCA dataset. (C) MOSTA dataset. (D) BARISTA dataset.

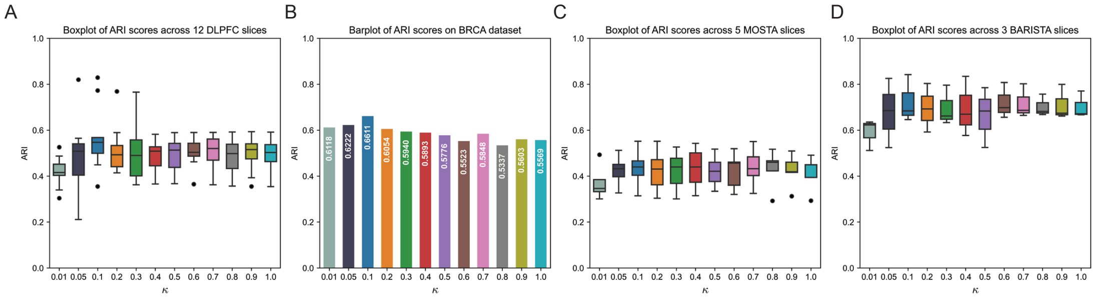

Fig S38 Performance of spCLUE with different values of κ on DLPFC (A), BRCA (B), MOSTA (C), and BARISTA (D) datasets.

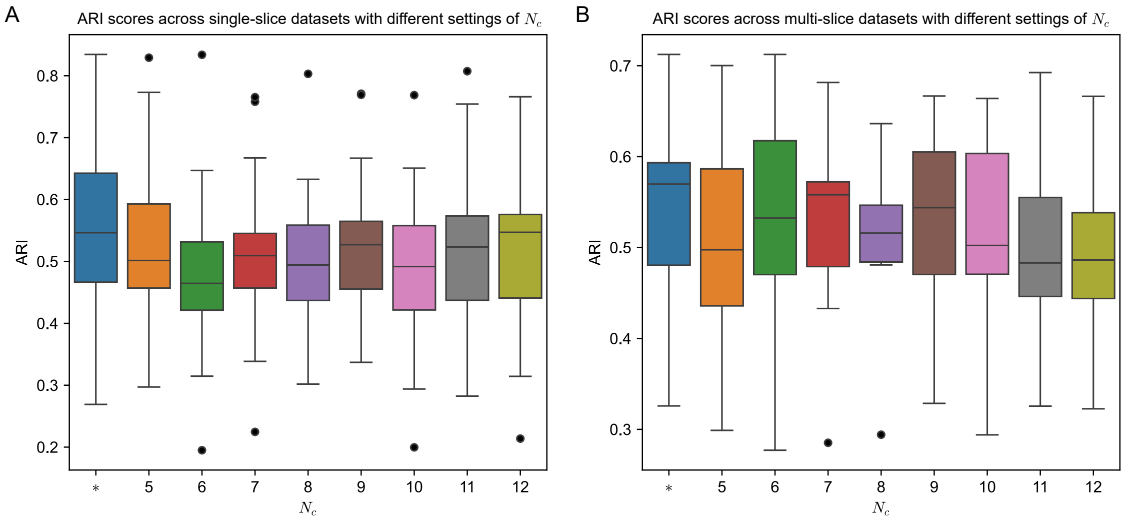

Fig S39 ARI scores of spCLUE with different settings of N_c across single-slice (A) or multi-slice (B) datasets. $*$ refers to the ARI scores when N_c is set to the known number tissue layers or cell types.

3 Supplementary Tables

Table S1: Comparison between spatial domain identification methods. Under the “Applicability” category, “Single-slice” and “Multi-slice” indicate whether the method can be applied to single-slice or multi-slice data for identifying spatial domains, while “Spot embedding” indicates whether the method simultaneously learns latent spot embeddings and identifies spatial domains. Under the “Methodology” category, “Deep learning” indicates whether the method is built on a deep learning framework; “Multi-view graph network” indicates whether the method leverages multiple graph views within the neural network; “Contrastive learning” indicates whether the method adopts a contrastive learning strategy; “Attention mechanism” indicates whether the method incorporates attention modules in its learning framework; and “Explicit batch effect removal” indicates whether the method includes procedures specifically designed to address batch effects. Gray cells indicate that the feature is not applicable. The above features are summarized as introduced in each method’s original publication. DGI: deep graph infomax (a contrastive learning approach that learns spot representations by encouraging agreement between real spot embeddings and a global summary of the graph, while distinguishing them from embeddings derived from a corrupted version of the graph).

Method	Year	Applicability			Methodology					
		Single-slice	Multi-slice	Spot embedding	Spatial information	Deep learning	Multi-view graph network	Contrastive learning	Attention mechanism	Explicit batch effect removal
Seurat	2018	√	√	√						√
Harmony	2019		√	√						√
BayesSpace	2021	√	√		√					√ (Use Harmony)
SpaGCN	2021	√			√	√				
STAGATE	2022	√	√ (Require aligned coordinates)	√	√	√			√ (Apply an attention module to the spatial graph to update spot embeddings)	
CCST	2022	√		√	√	√		√ (Use the DGI approach)		
SpaceFlow	2022	√		√	√	√		√ (Use the DGI approach)		
BASS	2022	√	√		√					√ (Use Harmony)
GraphST	2023	√	√ (Require aligned coordinates)	√	√	√		√ (Use a modified DGI approach)		
Spatial-MGCN	2023	√		√	√	√	√ (Construct an expression graph and a spatial graph)		√ (Apply an attention module to each view separately and combine embeddings through a weighted summation)	
STAligner	2023		√	√	√	√		√ (Used for batch effect removal)	√ (Apply an attention module to the spatial graph to update spot embeddings)	√ (Address batch effects by triplet contrastive learning, which compares anchor spots and corresponding positive spots and negative spots)
SEDR	2024	√	√	√	√	√				
MuCoST	2024	√		√	√	√	√ (Construct an expression graph, a spatial graph, and a shuffled graph)	√ (Formulated to maximize similarity of spot embeddings between the expression and spatial graphs, while minimizing the similarity between the spatial and shuffled graphs)		
STAIG	2025	√	√	√	√	√		√ (Formulated to compare positive and negative spot pairs in two augmented graphs generated from the original spatial graph)		√ (Address batch effects by limiting the selection of negative pairs to within the same slice)
spCLUE	2025	√	√	√	√	√	√ (Construct an expression graph and a spatial graph)	√ (Formulated as two complementary modules: the instance constrastive module encourages similarity of spot embeddings between the expression and spatial graph, and the clustering constrastive module enhances clustering signals in the embedding space)	√ (Apply an attention module to both the expression and spatial graphs to integrate spot embeddings from the two views)	√ (Remove batch effect using a batch prompting module)

Table S2: Datasets used for method evaluation.

Dataset	Platform	Organism	Tissue	Slice	Spot	Gene
DLPFC	10x Visium	Human	Dorsolateral pre-frontal cortex	151507	4226	33538
				151508	4226	33538
				151509	4384	33538
				151510	4634	33538
				151669	3661	33538
				151670	3498	33538
				151671	4110	33538
				151672	4015	33538
				151673	3639	33538
				151674	3673	33538
				151675	3592	33538
				151676	3460	33538
BRCA	10x Visium	Human	Breast tumor	/	3798	36601
MOB1	Slide-seqV2	Mouse	Olfactory bulb	/	20139	11750
MOB2	Stereo-seq	Mouse	Olfactory bulb	/	19109	14376
MOSTA	Stereo-seq	Mouse	Embryo	E1S1	5913	25568
				E2S1	5292	23756
				E2S2	4356	24107
				E2S3	5059	24238
				E2S4	5797	23398
BARISTA	BaristaSeq	Mouse	Cortex	slice1	1525	79
				slice2	2042	79
				slice3	1690	79

Table S3: ARI scores of spCLUE and the ablated versions. For each slice, the score in bold font represents the best method, and the score with * represents the second best method. "feat_spa" is short for "feature_spa", and "feat_expr" is short for "feature_expr".

Slice	spCLUE	spCLUE w/o cluster	spCLUE w/o instance	spCLUE w/o both	feat_spa	feat_expr
151507	**0.546**	0.432	0.361	0.481	0.545*	0.508
151508	**0.568**	0.421	0.48	0.383	0.508	0.53*
151509	0.552	0.423	0.418	0.411	0.566*	**0.592**
151510	0.449	**0.485**	0.397	0.467*	0.414	0.402
151669	**0.502**	0.351	0.317	0.331	0.353*	0.205
151670	**0.355**	0.321*	0.293	0.22	0.202	0.239
151671	0.829*	0.572	0.407	0.362	0.415	**0.833**
151672	**0.773**	0.526	0.607	0.423	0.46	0.745*
151673	**0.568**	0.519*	0.419	0.312	0.503	0.43
151674	**0.494**	0.249	0.424	0.272	0.488*	0.407
151675	**0.531**	0.499	0.384	0.35	0.422	0.5*
151676	**0.548**	0.408	0.353	0.421	0.543*	0.46
BRCA	**0.661**	0.464	0.535	0.564	0.548	0.632*
E1S1	0.269	0.259	0.282	**0.312**	0.279	0.282*
E2S1	0.403	0.388	0.299	0.406*	0.356	**0.414**
E2S2	**0.466**	0.412	0.328	0.451	0.387	0.464*
E2S3	**0.496**	0.363	0.465	0.447	0.464	0.468*
E2S4	0.44	0.447	**0.459**	0.414	0.416	0.457*
slice1	0.643	**0.712**	0.65	0.706*	0.66	0.499
slice2	**0.834**	0.791	0.72	0.797*	0.705	0.563
slice3	**0.647**	0.568	0.542	0.529	0.629*	0.462
mean	**0.551**	0.458	0.435	0.431	0.470	0.481*
median	**0.546**	0.432	0.418	0.414	0.464*	0.464

Table S4: Simulated datasets used for batch effect removal evaluation.

Simulated dataset	Reference dataset	No. gene	No. batch	No. domain	No. spot
BARISTAsim	BARISTA (slice1)	79	3	6	(1525,2042,1690)
DLPFCsim	Sample3 (151673)	12000	4	7	(3611, 3635, 3566, 3431)
MOSTAsim	MOSTA (E1S1)	12000	5	18	(5913, 5292, 4356, 5029, 5797)
